# Bacterial Contamination of Healthcare Students’ Mobile Phones: Impact of Specific Absorption Rate (SAR), Users’ Demographics and Device Characteristics on Bacterial Load

**DOI:** 10.3390/life13061349

**Published:** 2023-06-08

**Authors:** Massimo Maurici, Francesca Pica, Gian Loreto D’Alò, Domenico Cicciarella Modica, Alessandra Distefano, Margarida Gorjao, Maria Sofia Simonelli, Livio Serafinelli, Patrizia De Filippis

**Affiliations:** 1 Department of Biomedicine and Prevention, University of Rome Tor Vergata, 00133 Rome, Italy; maurici@med.uniroma2.it (M.M.); gianloretod@gmail.com (G.L.D.); alessandra.distefano@hotmail.it (A.D.); patrizia.de.filippis@uniroma2.it (P.D.F.); 2 Department of Experimental Medicine, University of Rome Tor Vergata, 00133 Rome, Italy; 3 District 6, Local Health Authority Roma 2, 00100 Rome, Italy; 4 District 4, Local Health Authority Roma 6, 00100 Rome, Italy; domenico.cicciarellamodica@gmail.com; 5 School of Hygiene and Preventive Medicine, University of Rome Tor Vergata, 00133 Rome, Italy; margarida.gorjao@students.uniroma2.eu (M.G.); mariasofia.simonelli@students.uniroma2.eu (M.S.S.); serafinelli.livio@gmail.com (L.S.)

**Keywords:** mobile phones, specific absorption rate (SAR), Staphylococci, Enterococci, Gram negative bacteria, healthcare students

## Abstract

We quantitatively and qualitatively evaluated the bacterial contamination of mobile phones (MPs) in relation to users’ demographics, habits, and device characteristics by administering questionnaires to 83 healthcare university students and sampling their MPs by following a cross-sectional design. The heterotrophic plate count (HPC) at 22 °C (HPC 22 °C) and 37 °C (HPC 37 °C), Enterococci, Gram-negative bacteria, and Staphylococci were evaluated. Higher bacterial loads were detected for HPC 37 °C and Staphylococci (416 and 442 CFU/dm^2^, respectively), followed by HPC 22 °C, Enterococci, and Gram-negative bacteria; the vast majority of samples were positive for HPC 37 °C, HPC 22 °C, and Staphylococci (98%), while Enterococci (66%) and Gram-negative bacteria (17%) were detected less frequently. A statistically significant positive correlation (r = 0.262, *p* < 0.02) was found between the European head specific absorption rate (SAR) and both HPC 37 °C and Staphylococci; Enterococci showed a strong, significant correlation with HPC 37 °C, HPC 22 °C, and Gram-negative bacteria (r = 0.633, 0.684, 0.884) and a moderate significant correlation with Staphylococci (r = 0.390). Significant differences were found between HPC 22 °C and the type of internship attendance, with higher loads for Medicine. Students with a daily internship attendance had higher HPC 22 °C levels than those attending <6 days/week. Our study showed that bacteria can survive on surfaces for long periods, depending on the user’s habits and the device’s characteristics.

## 1. Introduction

Mobile phones (MPs) are ubiquitous communication devices and are currently used in private as well as professional life. The estimated number of users in 2021 was 7.1 billion, and this number is projected to be 7.49 billion in 2025; 90% of young Europeans and Asians and 86–94% of individuals under 65 years old own mobile phones [1]. MPs are largely recognized as fomites, as their surfaces, touched by hands an average of two thousand times a day, are home to viable microorganisms [2,3,4]. 

In hospitals, MPs are widely used by healthcare workers (HCWs) and students of healthcare professions within and outside hospital wards, both as personal communication devices and useful tools for clinical practice [5,6,7,8]. This could reasonably produce an increased occurrence and transmission of healthcare-associated infections (HAIs) considering that they are potential carriers of microorganisms [2,9,10,11]. Most people, including healthcare workers (HCWs), are not aware that several bacteria rest on their devices during and after a patient’s visit; indeed, after contact with a contaminated surface, HCWs can transmit microorganisms to other clean surfaces and the skin [12,13,14,15,16,17]. 

Several studies have been conducted to explore microbial contamination on HWCs’ MPs; potentially pathogenic microorganisms were found on MP surfaces in 95% observational studies, especially in cases in which no particular attention was paid to cleaning and disinfecting such devices [18,19]. Drug-resistant bacteria have been found on HWCs’ MPs by several studies; these devices may thus facilitate the spread of antibiotic resistance both in and out of hospital settings [2,20].

HAIs significantly impact the health of hospitalized patients and are characterized by a growing epidemiological trend; they also have an important impact on the financial aspects of health management [18,21]. The Centers for Disease Control and Prevention (CDC) consider hand hygiene the most important practice in reducing the spread of pathogens in healthcare settings (https://www.cdc.gov/handhygiene/index.html accessed on 28 April 2023). Nevertheless, several studies have shown that awareness of the importance of hand hygiene is not adequately disseminated among health professionals, so compliance with good hand hygiene practices remains poor [6,22].

The occurrence of HAIs following cross-contamination through MPs has not been directly documented so far due to the lack of studies investigating this link [7,18]. However, given the levels and prevalence of surface contamination of MPs, several studies have sought to investigate the presence of predisposing factors for colonization by potentially pathogenic bacterial species. 

The bacterial loads found on the surface of MPs have been related to various factors, such as gender [15,19,23,24,25], education level [5,19,24,26], use of public transport [19,27], MP use habits [15,19], and MP cleaning habits [15,19,24,28], even though the findings are not univocal [29].

Recently, microbial growth on MPs was related to the electromagnetic radiation (EMR) emissions of the same MPs by considering the value of the devices’ specific absorption rate (SAR) as an independent variable [30]. MPs are complex electronic devices that receive and transmit EMR at the frequency range of radio frequencies [31]. EMR transfers energy on materials, causing both thermal and biological effects on unicellular and multicellular living organisms [30,32,33]. The heat generated by MP use and their placement in pockets contribute to the generation of the conditions for incubation, favoring the survival of microorganisms on their surface for a long time [29,34]. In addition, in 2011, the IARC/WHO classified radiofrequency electromagnetic fields as a Class 2B carcinogen [see urly.it/3tzh0].

Regulatory organizations of various countries around the world have established threshold levels for the EMR emissions of MPs, indicated through the SAR. The SAR is usually measured at the head and trunk level [30,33]. In the USA, Canada (FCC/ISED standard), and India, the maximum allowed level is an SAR of 1.6 W/kg, while in the EU (IEEE/IEC standard) the limit is 2.0 W/kg (European head SAR) [30,32,33,35]. 

We previously reported the results of a study in which the contamination of university healthcare students’ MPs was investigated in relation to their demographics and habits [19]. The present study aimed to extend our previous observations by evaluating, on a quantitative and qualitative level, the bacterial contamination of MPs of university students attending health degree courses as well as by implementing the specific analytical study methodology and in relation to other new variables, including the SAR, with a possibly critical impact on the observed phenomenon.

## 2. Materials and Methods

### 2.1. Study Sample

Eighty-three students from the University of Rome Tor Vergata in degree courses in healthcare professions (nursing, midwifery, and other degree courses) were enrolled in the study.

In order to estimate the sample size needed, we used the formula applicable to prevalence studies, as reported by Arya et al. [36]. On the basis of our previous experience, we considered a bacterial prevalence, in terms of samples positive for HCP 37 °C, of 96.2% (i.e., 114 out of 118 samples) [19], so the estimated sample size was 56 students. Overall, 83 MPs were included in the study. The prevalence of HPC 37 °C-positive samples was confirmed in the present study (98.8%, see Results section).

The project was approved by an independent ethical committee. Informed consent was obtained from each participant prior to enrollment after they received all the necessary information about the investigation’s purpose. All data were managed according to the current European guidelines and regulations, as reported previously [19].

### 2.2. Questionnaire Administration

Enrollment was voluntary and strictly anonymous. The students willing to participate in the study were asked to fill out a short questionnaire and provide their own MPs for microbiological analyses. The questionnaire included a demographic section and a specific section consisting of 11 items (see Table 1). Each completed questionnaire was assigned a progressive ID number for data recording and processing.

Ten enrollment sessions were performed in the period of 23 October 2019–19 February 2020 at the University soon after classes, and a maximum of 10 students of healthcare degree courses per session were enrolled. 

### 2.3. Microbiological Analysis

The MP surface sampling for microbiological analyses was performed in front of each MP’s owner (the student) in order to avoid cross-contamination. The collection of samples, the heterotrophic plate count (HPC) at 37 °C (HPC 37 °C) and 22 °C (HPC 22 °C), and the isolation and biochemical identification of Staphylococci, Enterococci, and Gram-negative bacteria were performed using the methods, kits, and reagents reported in our previous study [19].

### 2.4. Statistical Analysis

Data were recorded on an MS Excel^®^ worksheet. We performed a descriptive analysis of the students’ answers, expressing each variable and category as the absolute number and percentage. For microbiological results, we described quantitative variables both as the mean and standard deviation (SD) and as the median and interquartile range (Q1–Q3); we also performed stratification on the basis of the questionnaire’s variables (see Supplementary Material, Table S1). 

We performed qualitative analysis using R software v. 3.6.2 (The R Foundation, Vienna, Austria; https://www.r-project.org/). We built a heatmap to demonstrate differences across variables after dichotomizing 3 of the continuous variables (i.e., HPC 37 °C, HPC 22 °C, and total count of Staphylococci) by selecting a threshold of 100 CFU/dm^2^ [37]. For the single Staphylococcus species, the Gram-negative bacteria, and the Enterococci, we used the simple frequency of occurrence. 

We also built scatter plots with R expressing bacterial concentrations as the natural logarithm (ln) of CFU/ dm^2^; in the same plots, categorical variables were shown through the mean value and its confidence interval (IC) at 95%. For each categorical variable, we made 2 scatter plots, one comparing HPC at 37 °C with HPC at 22 °C and the other comparing Staphylococcal and Enterococcal loads.

We also built radar plots for each categorical variable with Excel^®^. The radars plots were built by standardizing each type of bacterial charge (i.e., HPC 37 °C, HPC 22 °C, Enterococci, Gram-negative bacteria, and Staphylococci) in values ranging from 0 to 1, with 0 = the minimum value for the specific charge (e.g., min HPC 37 °C = 0 CFU/dm^2^) and 1 = the maximum detected value for the specific charge (e.g., max HPC 37 °C = 2082 CFU/dm^2^). Standardized values were then averaged for each categorical variable. 

MP age values were divided into “low” and “high” categories according to the sample’s mean value (“low”: ≤mean; “high”: >mean), while the European head specific absorption rate (SAR) was divided into 3 categories (“low”, “medium”, and “high”), consistent with the trimodal distribution proposed by Di Lodovico et al. [30]. The data on the European head SAR were retrieved from the manufacturer’s declarations reported on the technical data sheets of the individual smartphone models used by the students. Briefly, the students were asked to report the MP’s model on the questionnaire, and the technical data sheet for each indicated model was retrieved by searching the internet (i.e., manufacturers’ websites, vendors’ websites).

Linear regression and Pearson’s correlation coefficient were used to check the relationship between continuous variables. The ANOVA test was applied to compare mean CFUs (HPC at 22 °C, HPC at 37 °C, Enterococci, Staphylococci, and Gram-negative bacteria) among grouping (categorical) variables (see Table 1). We eliminated outliers with CFU values greater than 3 SD. When significant differences were observed, a Bonferroni or Games–Howell (as assessed by Levene’s test) post hoc comparison was performed. Quantitative analyses were carried out with SPSS^®^ ver. 22.0. We considered *p*-values less than 0.05 statistically significant.

## 3. Results

### 3.1. Study Population

The demographic characteristics of the 83 participants in the study and the overall results of our survey are shown in Table 1. The composition of the final sample, in terms of basic demographic characteristics (such as gender and age), was very similar to those in the various healthcare degree courses. 

The average age of the students in the sample was 21.29 years, with a maximum of 35 and a minimum of 19; the age of the majority of participants (78.3%) was equal to or less than the average age of the sample.

The type of internship attendance was in medicine and surgery wards for the majority of the enrolled students. More than three-quarters of the students (77.1%) attended the wards and/or outpatient clinics 6 days/week, while the others reported an attendance of less than or equal to 5 days/week. Surgical wards were the most frequently visited (51.8%), followed by medical wards (39.8%); a low percentage of students attended outpatient clinics (3.6%) and intensive care units (2.4%).

The nursing degree course was by far the most represented (71.1%), whereas the midwifery degree course and the other healthcare degree courses together accounted for less than 30% of the sample (15.7% and 13.3%, respectively). All the participating students were in the second year of their degree courses. 

**Figure 1:**
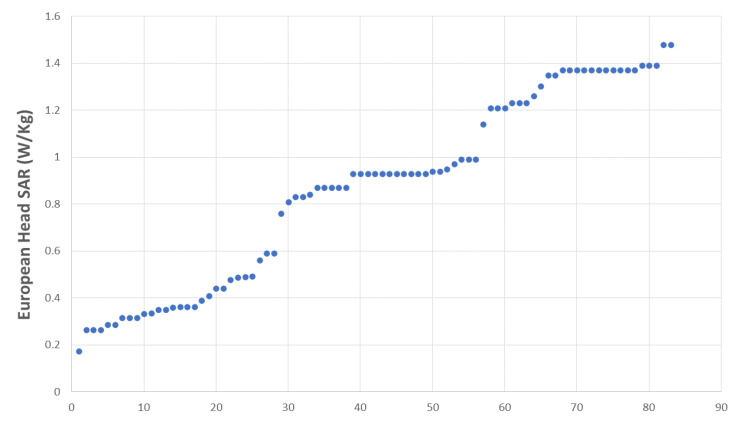
European head SAR distribution across sample.

Most of the students (89.2%) declared that they used their MPs during their training (Table 1). The specific absorption rates (SAR) of all the tested MPs are shown in Figure 1, and 60.2% of them were over the mean European head SAR, i.e., 0.87 W/Kg (Table 1). About 55% of the MPs had an age below the mean MP age, i.e., 18.78 months. 

With regard to the cleaning habits of the touchscreen, only 13.3% of the students had never cleaned their phones, and the others used different cleaning methods. The use of disinfectants (gels, sprays, alcohol-based products, or a combination of these) was declared by 32.5% of the students; 31.3% used water-based products/detergents, including soap and water or wet wipes, and 22.9% used “dry” methods (glass cloths, reusable wipes for glasses, and paper tissues).

Concerning the type of cover used to protect their phones, most of the students (88%) had a silicone case, 3.6% had a “flip-cover”, and 8.4% did not use a cover.

For the usual means of transport used, fewer students reported using public transport (42.2%) than private transport (57.8%). Moreover, focusing on the sampling day, students favored the use of private vehicles (63.9%); only 33.7% of them used public transport, and the remaining 2.4% used both.

Most of the students (89.2%) used their phones in the hospital wards; however, 77.1% of them declared that they never used their MPs while wearing gloves. On the other hand, 22.9% reported using their MPs while wearing gloves, with about half of them changing the gloves after using the MP. 

The European head SAR distribution was found to be trimodal (Figure 1), with values for the three categories ranging from 0.17 to 0.59 W/kg (low), from 0.76 to 0.99 W/kg (medium), and from 1.14 to 1.488 W/kg (high).

### 3.2. Microbiological Results

**Figure 2:**
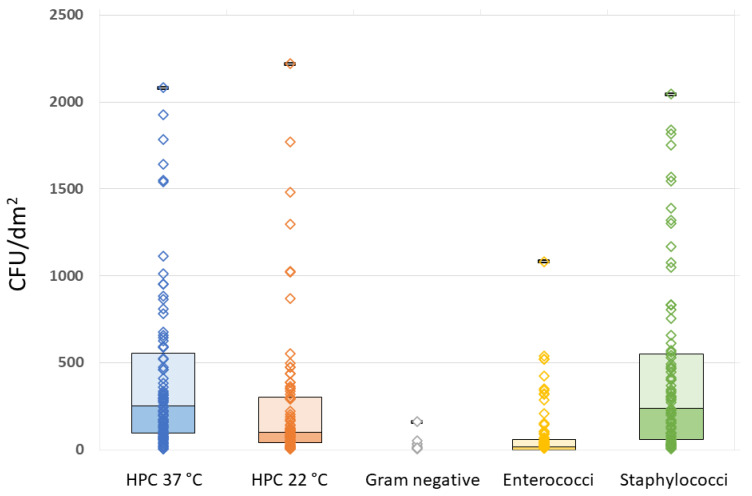
Raw data plots of HPC 37 °C, HPC 22 °C, Gram-negative, Enterococcal, and Staphylococcal plate counts.

All the analyzed MPs showed some degree of bacterial contamination, although with wide variability in both quantitative and qualitative terms. The quantitative results of the microbiological analyses and related raw data for HPC at 37 °C and 22 °C, Gram-negative organisms, Enterococci, and Staphylococci are summarized in Table 2 and Figure 2.

It can be seen that Staphylococci outnumbered all the other microorganisms (Table 2). The full results for HPC at 22 °C and 37 °C, Gram-negative bacteria, Enterococci, and Staphylococci in relation to the questionnaire variables are reported in the Supplementary Material, Table S1.

Regarding the identification of Staphylococcal isolates, in this study, *S*. *epidermidis* was the most common species isolated (73/83, 87.95% of MPs), followed by *S*. *aureus* and *S*. *warneri* (both 10/83, 12.05%), *S*. *cohnii cohnii* (2/83, 2.4%), *S*. *haemolyticus*, and *S*. *capitis* (both 1/83, 1.2%); *Micrococcus* spp. were also observed on 54.2% of MPs (45/83). We found a maximum of four different Staphylococcal species on the same MP. This occurred in a single case; 12 MPs hosted three different species, 35 MPs hosted two species, and only one species was found on the remaining 33 MPs.

**Figure 3:**
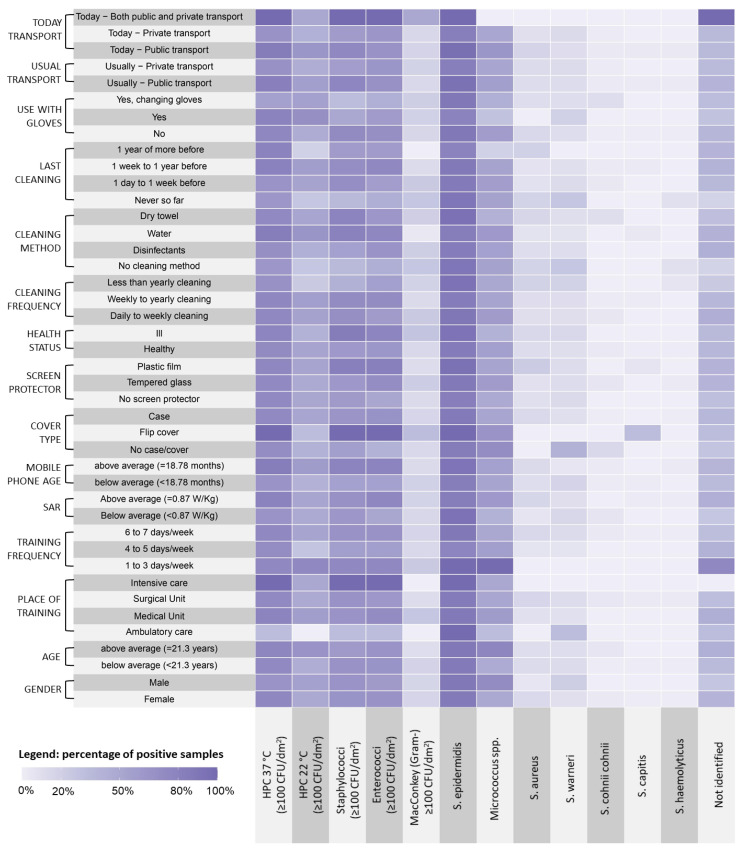
Distribution of the microbiological findings related to students’ demographic characteristics and habits.

Gram-negative bacteria were detected on 14 MPs (16.87% of the total), and on one of those, two different strains were isolated. Among the 14 isolated Gram-negative strains, *Enterobacter* spp., *Pasteurella* spp., *Pseudomonas* spp., and *Proteus* spp. were identified. A quantitative representation of the different bacterial strains in relation to the considered variables is shown in Figure 3. In particular, at HPC 37 °C, Staphylococci and Enterococci were well-represented in relation to all the tested variables, with higher loads found on MPs of students who used flip covers, attended intensive care units, and used public transport on the day of sampling. *S*. *epidermidis* was the more represented Staphylococcal species in the tested samples, and *S*. *capitis* was associated with the use of a flip cover.

**Figure 4:**
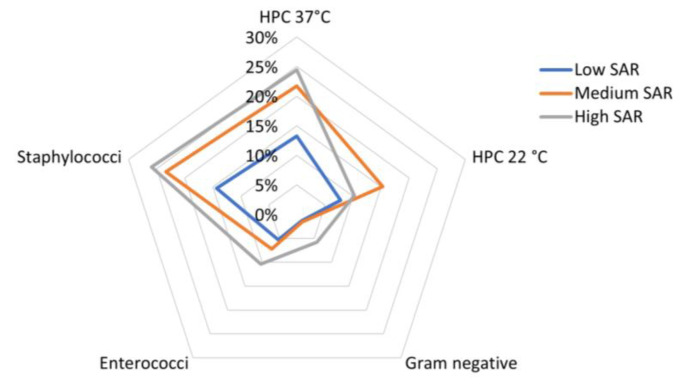
Radar plot showing standardized mean bacterial load detected for each class of European head SAR in the sampled mobile phones.

The variability of the bacterial load across the three SAR groups is presented in a radar plot in Figure 4.

From a visual inspection of the plot, it appears that high levels of HPC 37 °C and Staphylococci were both correlated with medium or high SAR values (the mean bacterial load was about 25% of the maximum detected bacterial load in HPC 37 °C and Staphylococci, respectively); however, the Enterococci load increased together with the SAR values, and the Gram-negative load was relatively high in samples from MPs with high SAR values.

The radar plots for all the questionnaire’s characteristics are provided in the Supplementary Material (Figures S1–S15). In addition, scatter plots showing the distribution of the mean bacterial charges in terms of Staphylococci vs. Enterococci and HPC 37 °C vs. HPC 22 °C across the user demographics and device characteristics, are reported in the Supplementary Material (Figures S16–S45).

A small, statistically significant correlation was verified between the SAR and HPC at 37 °C and Staphylococci (*p* = 0.017 and 0.018, r = 0.262 and 0.262, respectively). Staphylococci correlated with all the variables tested except Gram-negative bacteria and phone age (Table 3). Enterococci showed a strong correlation with HPC at 37 °C, HPC at 22 °C, and Gram-negative bacteria (r = 0.633, 0.684, and 0.884) and a moderate correlation with Staphylococci (r = 0.390); all correlations were statistically significant. Isolating *S. aureus*, the correlation with Enterococci was always statistically significant (*p* = 0.012) but much stronger (r = 0.866).

As verified using one-way ANOVA and post hoc tests, statistically significant differences in CFU/dm^2^ averages were found between HPC at 22 °C and type of internship attendance, i.e., outpatient clinic vs. medicine and surgery wards (42.6 CFU/ dm^2^ vs. 174.6 and 140.3 CFU/ dm^2^, respectively). In addition, those who attended the wards and/or outpatient clinics every day had higher concentrations of microorganisms (HPC 22 °C) than those who attended fewer than 6 days a week (156.7 CFU/ dm^2^ vs. 76.6 CFU/ dm^2^, respectively). Those who used public transport on the day of sampling had a higher concentration of CFU/ dm^2^ than those who used private transport (206 vs. 117.6 CFU/dm^2^, respectively; *p* = 0.013).

With regard to the presence of Gram-negative bacteria, it was observed that those who used MPs with gloves and changed them had a lower CFU value than those who did not use gloves (32.1 vs. 92.6 CFU/dm^2^; *p* = 0.006).

## 4. Discussion

The present study investigated the bacterial contamination of healthcare students’ MPs at the University of Rome Tor Vergata in relation to the user demographics and device characteristics, including the specific absorption rate (SAR). The results of the study confirmed that the MPs used by healthcare students in hospital settings hosted saprophytic and pathogenic bacteria, in agreement with data reported in the literature [2,3,4]. 

Staphylococci were the most represented bacteria, suggesting the possibility that the contamination of MPs was mostly due to the normal skin saprophytes [38]. *S*. *aureus*, currently considered a marker of defective hygienic quality for surfaces in the hospital context, was less represented in our samples (only 10 isolated organisms) compared with the other Staphylococcal strains (e.g., *S*. *epidermidis* has been isolated from 73 samples); however, its pathogenicity together with its tendency to develop antibiotic resistance requires that even low levels of contamination are interpreted with caution [39]. At the same time, the relevant presence in our samples of *S*. *epidermidis* and other less frequently detected staphylococcal strains (i.e., *S*. *warneri*, *S*. *cohnii cohnii*, *S*. *capitis*, and *S*. *haemolyticus*) also deserves consideration because of their etiological role in several human diseases, especially in hospital settings and in immunocompromised patients [40]. It is worth mentioning that *S*. *epidermidis* shows an elevated tendency to form biofilms on both biological and artificial surfaces; its resistance on MP touchscreen surfaces allows it to be easily carried throughout hospital settings [41]. Together with Staphylococci, *Micrococcus* spp. were also found to be highly represented on the tested MPs (detected in 45/83 MPs), consistent with their origin from saprophytic cutaneous microbiota, followed by oral, pulmonary, and intestinal ones [42]. By contrast, the Gram-negative opportunistic pathogens identified, namely *Enterobacter* spp., *Pasteurella* spp., *Proteus* spp., and *Pseudomonas* spp., although not highly represented in our sample (detected in only 14/83 MPs), were found to be associated with the highest CFU/dm^2^ values of the other bacterial strains. The fact that, in our investigation, the co-presence of Gram-negative and Gram positive-microorganisms was associated with the highest contamination level on MPs seems to indicate the presence of Gram-negative bacteria as a proxy of relevant MP contamination.

Considering that microorganisms belonging to the normal skin microbiota and environmental microorganisms could contaminate these devices, our study used standard methods of environmental microbiology, allowing us to evaluate mesophilic and psychrophilic organisms through the determination of HPC 22 °C and HPC 37 °C [19]. The mean and median numbers of CFU/dm^2^ detected at 37 °C were slightly higher than those at 22 °C, but the minimum and maximum levels were almost equal. This figure is not surprising if we consider that MPs are subject to continuous changes in temperature (heating and cooling) in relation to the type of device, the frequency of their use, and the methods of coverage and storage. In particular, Figure 4 shows that high levels of HPC 37 °C and Staphylococci were both correlated with medium or high SAR values; however, the Enterococci load increased together with SAR values, and the Gram-negative load was relatively high in samples from MPs with high SAR values. These findings are in line with previous observations that the heat generated by MPs due to their use and placement into the pockets contributes to the generation of the optimal conditions for incubation, favoring the survival of microorganisms on their surface for a long time [29,34]. 

Further analyses evidenced a small but statistically significant correlation between SAR and HPC at 37 °C and Staphylococci (*p* = 0.017 and 0.018, r = 0.262 and 0.262, respectively). Moreover, Staphylococci correlated with all the variables tested except Gram-negative bacteria and phone age (Table 3). By contrast, Enterococci showed a strong correlation with HPC at 22 °C, HPC at 37 °C, and Gram negative-bacteria (r = 0.633, 0.684, and 0.884) and a moderate correlation with Staphylococci (r = 0.390); all correlations were statistically significant. Isolating *S*. *aureus*, the correlation with Enterococci was always statistically significant (*p* = 0.012) but much stronger (r = 0.866). These last observations are not surprising considering that the co-presence of Enterococci and Gram-negative bacteria, although not predominant, could represent an index of poor individual hygiene habits or incidental contamination by the fecal route [43].

The MPs of the students attending the medicine and surgery wards were more contaminated than those of students attending the outpatient clinic and intensive care units. In addition, the MPs of students attending internships every day compared with those of students with a lower weekly attendance had higher concentrations of microorganisms (i.e., 156.7 vs. 76.6 CFU/dm^2^). MPs of students who used public transport had a higher concentration of HPC 22 °C, which was at the limits of statistical significance (*p* = 0.058), and those of students who used public transport on the day of the sampling had a significantly higher concentration of HPC 22 °C (206.02 vs. 117.55 CFU/dm^2^, *p* = 0.013). All these findings indicate that more continuous hospital ward attendance over time with inpatients as well as the use of public transport, which both lead to closer person-to-person contact, represent two important factors increasing the level of MPs contamination.

Nonetheless, in relation to the presence of Enterococci, we observed that MPs of students who used MPs with gloves and changed them after use were characterized by lower CFU/dm^2^ values than MPs of those who did not use gloves (32.1 vs. 92.6 CFU/dm^2^, *p* < 0.006), highlighting the fact that the correct use of gloves and adherence to the handwashing guidelines are crucial in preventing the intra-hospital circulation of potential pathogenic microorganisms [44,45].

Considering Staphylococci, we found that MPs of students with internships in the medical and surgical wards had a higher bacterial load than those of students attending the outpatient clinic (*p* = 0.017 medical and *p* = 0.002 surgical, 87 CFU vs. 291 medical and 325 surgical); in addition, the use of flip covers was statistically significantly correlated with higher CFU/dm^2^ with respect to the use of other types of cover (about three times higher bacterial load).

In conclusion, our results confirm that bacteria can survive on inanimate surfaces for extended time periods [46], realistically in multiple polymicrobial associations in biofilms, through a form of cooperative group behavior [47]. It is well known that under these conditions, they could live and multiply while staying protected from environmental stresses (i.e., desiccation and shear forces) and/or external attacks (i.e., the host’s immune system and antimicrobial agents) [47]. Furthermore, interspecies relationships are not fixed but can change depending on the prevailing environmental conditions [48]. In this regard, herein, we report evidence that the SAR and selected demographic and behavioral characteristics of the MP’s owner significantly impact the number and type of microorganisms living on the surface of MPs.

For these reasons, it is mandatory, especially in healthcare institutions, to focus on specific hygienic procedures, including the proper use of disposable gloves and hand washing. These procedures should be implemented before and after physical contact with patients. Moreover, cleaning MPs before and after hospital working shifts should be advised in order to reduce the risk of transferring pathogenic and drug-resistant bacteria from the hospital to the community [49]. 

Among this study’s limitations, it is worth mentioning that the data collected by the questionnaires could be subject to reporting bias and/or recall bias, especially those related to smartphone age, cleaning frequency, last cleaning, and means of transport; we attempted to address the potential bias by using wide categories and ensuring the anonymity of the questionnaire. Moreover, our results did not allow us to conduct inferential analyses in relation to single bacterial species and Gram-negative bacteria, as the study sample was not calculated on their low prevalence. Another study limitation is that we did not look for the presence of viruses and fungi; limited literature has been published on this topic [2], and exploring the relationship between bacterial and other microorganisms, together with user habits and device characteristics, could be a matter of interest for future research. Larger future studies or meta-analyses could be able to identify user demographics and device characteristics related to the presence of each microbial species.

Finally, it is of note that our study relates to the time period immediately preceding the onset of the COVID-19 pandemic, which most likely profoundly changed the population’s hygienic habits, both in hospital settings and communities. Therefore, it would be interesting to repropose the study presented herein in current times, during the post-COVID era, to ascertain if, how, and how much things have changed in this specific area. 

## Figures and Tables

**Table 1:** Demographic, behavioral, and MP characteristics of the sample.

Variables	Values	n	%
Degree course	Nursing	59	71.1
	Obstetrics	13	15.7
	Other	11	13.2
Gender	Male	18	21.7
	Female	65	78.3
Mean students age (21.29 years)	Below average	65	78.3
	Above average	18	21.7
Type of internship ward *	Ambulatory care	3	3.6
	Medical ward	33	39.8
	Surgical ward	43	51.8
	Intensive care	2	2.4
Weekly internship attendance (in days) *	1–3	4	4.8
	4–5	14	16.9
	6–7	64	77.1
Use of MP during training *	No	8	9.6
	Yes	74	89.2
Mean European head SAR (0.87 W/Kg)	Below average	33	39.8
	Above average	50	60.2
Mean MP age (18.78 months)	Below average	45	54.2
	Above average	38	45.8
Type of MP cover	None	7	8.4
	Flip-cover	3	3.6
	Case	73	88
Type of touchscreen protective film	None	31	37.3
	Glass	35	42.2
	Plastic	17	20.5
Acute illness ongoing *	No	68	81.9
	Yes	14	16.9
Frequency of MP cleaning	Up to 1/week	20	24.1
	From 1/8 days to 1/11 months	47	56.6
	1/year or never	16	19.3
Method of MP cleaning	None	11	13.3
	Disinfectants	27	32.5
	Water/detergents	26	31.3
	Dry towels	19	22.9
Last cleaning performed *	Never	11	13.3
	From 1 day to 7 days ago	24	28.9
	From 8 days to 11 months ago	41	49.4
	1 year or more ago	5	6
Use of MP with gloves	No	64	77.1
	Yes	10	12
	Yes, but I change gloves soon after	9	10.9
Usual means of transport	Public	35	42.2
	Private	48	57.8
Means of transport on the day of sampling	Public	28	33.7
	Private	53	63.9
	Both	2	2.4

* Not all respondents gave a valid answer to the item. The indicated percentages refer to the total sample.

**Table 2:** Quantitative evaluation of detectable microorganisms.

		HPC 37 °C (CFU/dm^2^)	HPC 22 °C (CFU/dm^2^)	Gram-neg. CFU/dm^2^	Enterococci (CFU/dm^2^)	Staphylococci (CFU/dm^2^)
N	Detectable	82	81	14	55	81
	Not detectable	1	2	69	28	2
Mean		416.16	253.24	27.79	124.46	442.49
Median		263.59	105.30	10.41	43.04	272.03
Std. Deviation		466.53	398.98	41.00	194.83	505.09
Range		2076.77	2217.01	153.90	1072.66	2035.88
Minimum		5.21	5.21	8.33	8.89	8.33
Maximum		2081.98	2222.22	162.23	1081.55	2044.21

**Table 3:** Pearson correlations (r) for main study variables.

	European Head SAR (W/Kg)	HPC 37 °C (CFU/dm^2^)	HPC 22 °C (CFU/dm^2^)	Gram-neg. (CFU/dm^2^)	Enterococci (CFU/dm^2^)	Staphylococci (CFU/dm^2^)	MP Age in Months
HPC 37 °C (CFU/dm^2^)	0.262 *		0.840 **	0.658 *	0.633 **	0.727 **	−0.055
HPC 22 °C (CFU/dm^2^)	0.099	0.840 **		0.733 **	0.684 **	0.716 **	−0.065
Gram-neg. (CFU/dm^2^)	0.260	0.658 *	0.733 **		0.884 **	0.436	−0.151
Enterococci (CFU/dm^2^)	0.108	0.633 **	0.684 **	0.884 **		0.390 **	−0.129
Staphylococci (CFU/dm^2^)	0.262 *	0.727 **	0.716 **	0.436	0.390 **		0.021
MP age in months	−0.110	−0.055	−0.065	−0.151	−0.129	0.021	

* Statistical significance set at the 0.05 level (two-tailed). ** Statistical significance set at the 0.01 level (2-tailed).

## Data Availability

The data presented in this study are available upon reasonable request from the corresponding author; the data are not publicly available due to privacy reasons.

## Supplementary Materials

The following supporting information can be downloaded at: https://www.mdpi.com/article/10.3390/life13061349/s1, Figures S1–S15: radar plots showing the bacterial charges across the 15 selected user demographics and device characteristics; Figures S16–S30: scatter plots comparing mean HPC 22 °C and HPC 37 °C across the selected variables; Figures S31–S45: scatter plots comparing mean Staphylococci and Enterococci charges across the selected variables; Table S1: full results for HPC 22 °C and HPC at 37 °C, Gram-negative organisms, Enterococci, and Staphylococci in relation to questionnaire variables.
