# Supplementary material for: Bacterial Contamination of Healthcare Students’ Mobile Phones: Impact of Specific Absorption Rate (SAR), Users’ Demographics and Device Characteristics on Bacterial Load
Source: Life (Basel). 2023 Jun 8;13(6):1349. doi: 10.3390/life13061349 (PMC10301942; doi:10.3390/life13061349)

BACTERIAL CONTAMINATION OF HEALTHCARE STUDENTS’ MOBILE PHONES: IMPACT OF SPECIFIC ABSORPTION RATE (SAR), USERS’ DEMOGRAPHICS AND DEVICE CHARACTERISTICS ON BACTERIAL CHARGES.

Maurici M, Pica F, D’Alò GL, Cicciarella Modica D, Distefano A, Gorjao M, Simonelli MS, Serafinelli L, De Filippis P.

Supplementary Material

Table of contents

TABLE S1: FULL RESULTS FOR HPC AT 37 °C, HPC 22 °C, GRAM NEGATIVE ORGANISMS OR ENTERICS, ENTEROCOCCI AND STAPHYLOCOCCI IN RELATION TO QUESTIONNAIRE’ VARIABLES. 3

RADAR PLOTS SHOWING THE BACTERIAL CHARGES ACROSS THE 15 SELECTED USER’S DEMOGRAPHICS AND DEVICES CHARACTERISTICS 8

- Figure S1: Radar plot for Gender 8
- Figure S2: Radar plot for User’s Age 8
- Figure S3: Radar plot for Place of training 9
- Figure S4: Radar plot for Training frequency 9
- Figure S5: Radar plot for European Head SAR 10
- Figure S6: Radar plot for Smartphone age 10
- Figure S7: Radar plot for Cover type 11
- Figure S8: Radar plot for Screen protector 11
- Figure S9: Radar plot for Health status 12
- Figure S10: Radar plot for Cleaning frequency 12
- Figure S11: Radar plot for Cleaning method 13
- Figure S12: Radar plot for Last cleaning 13
- Figure S13: Radar plot for Use with gloves 14
- Figure S14: Radar plot for Usual means of transport 14
- Figure S15: Radar plot for Means of transport in the day of sampling 15

SCATTER PLOTS COMPARING MEAN HPCS 37 °C AND HPCS 22 °C ACROSS THE SELECTED VARIABLES 16

Figure S16: HPCs 37 °C and HPCs 22 °C based on Age 16
Figure S17: HPCs 37 °C and HPCs 22 °C based on Cleaning frequency 17
Figure S18: HPCs 37 °C and HPCs 22 °C based on Cleaning method 18
Figure S19: HPCs 37 °C and HPCs 22 °C based on Cover type 19
Figure S20: HPCs 37 °C and HPCs 22 °C based on European Head SAR (W/kg) 20
Figure S21: HPCs 37 °C and HPCs 22 °C based on Gender 21
Figure S22: HPCs 37 °C and HPCs 22 °C based on Health Status 22
Figure S23: HPCs 37 °C and HPCs 22 °C based on Last Cleaning 23
Figure S24: HPCs 37 °C and HPCs 22 °C based on Means of transport in the day of sampling 24
Figure S25: HPCs 37 °C and HPCs 22 °C based on Place of training 25
Figure S26: HPCs 37 °C and HPCs 22 °C based on Screen protector 26
Figure S27: HPCs 37 °C and HPCs 22 °C based on Smartphone age 27
Figure S28: HPCs 37 °C and HPCs 22 °C based on Training frequency 28
Figure S29: HPCs 37 °C and HPCs 22 °C based on Use with gloves 29
Figure S30: HPCs 37 °C and HPCs 22 °C based on Usual mean of transport 30

SCATTER PLOTS COMPARING MEAN STAPHYLOCOCCI AND ENTEROCOCCI CHARGES ACROSS THE SELECTED VARIABLES 31

Figure S31: mean Staphylococci and Enterococci charges based on Age 31
Figure S32: mean Staphylococci and Enterococci charges based on Cleaning frequency 32
Figure S33: mean Staphylococci and Enterococci charges based on Cleaning method 33
Figure S34: mean Staphylococci and Enterococci charges based on Cover type 34
Figure S35: mean Staphylococci and Enterococci charges based on European Head SAR 35
Figure S36: mean Staphylococci and Enterococci charges based on Gender 36
Figure S37: mean Staphylococci and Enterococci charges based on Health Status 37
Figure S38: mean Staphylococci and Enterococci charges based on Last Cleaning 38
Figure S39: mean Staphylococci and Enterococci charges based on Means of transport in the day of sampling 39
Figure S40: mean Staphylococci and Enterococci charges based on Place of training 40
Figure S41: mean Staphylococci and Enterococci charges based on Screen protector 41
Figure S42: mean Staphylococci and Enterococci charges based on Smartphone age 42
Figure S43: mean Staphylococci and Enterococci charges based on Training frequency 43
Figure S44: mean Staphylococci and Enterococci charges based on Use with gloves 44
Figure S45: mean Staphylococci and Enterococci charges based on Usual mean of transport 45

Table S1: Full results for HPC at 37 °C, HPC 22 °C, Gram negative organisms or enterics, Enterococci and Staphylococci in relation to questionnaire' variables.

			HPC 37°C (CFU/dm^2)	HPC 22°C (CFU/dm^2)	Gram negative (CFU/dm^2)	Enterococci (CFU/dm^2)	Staphylococci (CFU/dm^2)
**Whole sample**	**(n=83)**	**Mean**	411	247	5	82	432
		**Median**	253	100	0	19	238
		**SD**	466	396	19	169	504
		**Q1**	88	42	0	0	60
		**Q3**	589	302	0	65	562
**Gender**	**Female**	**Mean**	391	235	6	82	420
	**(n=65)**	**Median**	274	83	0	18	238
		**SD**	442	402	22	175	499
		**Q1**	105	26	0	0	62
		**Q3**	522	296	0	66	536
	**Male**	**Mean**	485	292	2	84	476
	**(n=18)**	**Median**	214	106	0	28	221
		**SD**	553	380	4	151	530
		**Q1**	76	71	0	0	57
		**Q3**	888	335	0	57	858
**Age (mean = 21,29y)**	**Below average**	**Mean**	407	231	5	90	426
	**(n=65)**	**Median**	228	83	0	25	238
		**SD**	475	364	21	185	479
		**Q1**	88	26	0	0	60
		**Q3**	555	296	0	61	567
	**Above average**	**Mean**	427	305	3	55	453
	**(n=18)**	**Median**	320	152	0	16	222
		**SD**	443	503	13	89	600
		**Q1**	118	69	0	0	50
		**Q3**	630	351	0	92	578
**Study course**	**Nursing**	**Mean**	385	265	5	95	416
	**(n=59)**	**Median**	206	112	0	19	213
		**SD**	479	374	22	191	506
		**Q1**	88	54	0	0	60
		**Q3**	462	352	0	70	530
	**Obstetrics**	**Mean**	369	97	1	54	323
	**n=(13)**	**Median**	321	43	0	18	301
		**SD**	284	128	2	90	284
		**Q1**	163	18	0	0	38
		**Q3**	607	140	0	53	509
	**Other**	**Mean**	599	331	7	50	645
	**(n=11)**	**Median**	595	82	0	19	405
		**SD**	554	644	16	93	658
		**Q1**	77	32	0	0	95
		**Q3**	867	338	9	43	1051
**Place of training**	**Ambulatory care**	**Mean**	255	43	0	11	88
	**(n=3)**	**Median**	77	42	0	0	95
		**SD**	338	32	0	20	49
		**Q1**	44	11	0	0	35
		**Q3**			0		
	**Medical Unit**	**Mean**	482	305	10	122	490

	**(n=33)**	**Median**	281	121	0	30	287
		**SD**	538	488	30	230	553
		**Q1**	116	56	0	0	49
		**Q3**	798	354	8	89	779
	**Surgical Unit (n=43)**	**Mean**	371	216	1	55	408
		**Median**	228	95	0	17	292
		**SD**	423	326	4	104	472
		**Q1**	88	43	0	0	60
		**Q3**	520	293	0	44	530
	**Intensive care (n=2)**	**Mean**	279	66	0	40	179
		**Median**	279	66	0	40	179
		**SD**	112	65	0	42	83
		**Q1**	200	20	0	11	120
		**Q3**	.	.	0	.	.
**Training frequency**	**1 to 3 days/week (n=4)**	**Mean**	607	224	3	28	922
		**Median**	703	219	0	12	942
		**SD**	388	196	5	40	701
		**Q1**	200	40	0	3	246
		**Q3**	919	413	8	68	1577
	**4 to 5 days/week (n=14)**	**Mean**	393	230	4	56	344
		**Median**	272	58	0	19	210
		**SD**	506	579	14	106	474
		**Q1**	67	17	0	0	55
		**Q3**	506	151	0	37	446
	**6 to 7 days/week (n=64)**	**Mean**	394	243	5	88	406
		**Median**	225	110	0	18	227
		**SD**	464	356	21	182	478
		**Q1**	91	49	0	0	51
		**Q3**	509	314	0	66	521
**Smartphone age (mean 18,78m)**	**Below average (n=45)**	**Mean**	385	246	7	94	359
		**Median**	156	82	0	14	152
		**SD**	504	442	26	201	459
		**Q1**	76	29	0	0	34
		**Q3**	497	257	0	50	506
	**Above average (n=38)**	**Mean**	443	249	2	69	518
		**Median**	294	118	0	24	302
		**SD**	421	339	5	122	546
		**Q1**	169	44	0	10	148
		**Q3**	602	343	0	67	683
**Cleaning method**	**No cleaning method (n=11)**	**Mean**	630	378	16	152	470
		**Median**	144	78	0	0	92
		**SD**	774	639	49	333	662
		**Q1**	63	11	0	0	33
		**Q3**	1552	552	8	91	804
	**Disinfectants (n=27)**	**Mean**	322	206	5	65	358
		**Median**	222	75	0	18	158
		**SD**	396	443	12	113	515
		**Q1**	77	18	0	0	29
		**Q3**	383	166	0	43	417
	**Water (n=26)**	**Mean**	398	248	0	64	525
		**Median**	291	120	0	16	320
		**SD**	362	272	2	119	505
		**Q1**	132	54	0	7	155
		**Q3**	662	344	0	57	832
	**Dry towel**	**Mean**	429	230	3	92	387

	(n=19)	Median	281	114	0	32	303
		SD	453	296	8	162	389
		Q1	77	61	0	0	133
		Q3	589	357	0	108	481
Cleaning frequency	Daily to weekly cleaning (n=20)	Mean	442	270	7	84	401
		Median	349	119	0	23	319
		SD	441	480	15	147	420
		Q1	101	56	0	0	80
		Q3	600	333	6	81	531
	Weekly to yearly cleaning (n=47)	Mean	367	228	2	73	450
		Median	217	114	0	25	227
		SD	397	294	4	126	524
		Q1	100	43	0	0	60
		Q3	589	302	0	66	571
	Less than yearly cleaning (n=16)	Mean	502	275	11	108	419
		Median	227	58	0	10	98
		SD	664	545	40	280	564
		Q1	64	20	0	0	34
		Q3	731	106	0	44	648
Last cleaning	Never so far (n=11)	Mean	630	378	16	152	470
		Median	144	78	0	0	92
		SD	774	639	49	333	662
		Q1	63	11	0	0	33
		Q3	1552	552	8	91	804
	1 day to 1 week before (n=24)	Mean	388	236	6	73	376
		Median	314	109	0	19	282
		SD	429	444	14	136	406
		Q1	71	38	0	0	56
		Q3	511	313	6	62	531
	1 week to 1 year before (n=41)	Mean	391	242	1	79	470
		Median	244	114	0	30	287
		SD	402	303	4	131	535
		Q1	109	43	0	10	95
		Q3	590	357	0	70	571
	1 year of more before (n=5)	Mean	217	56	0	7	286
		Median	284	47	0	9	213
		SD	116	35	0	6	296
		Q1	102	31	0	0	15
		Q3	299	84	0	12	594
Cover type	No case/cover (n=7)	Mean	338	235	1	78	401
		Median	312	32	0	0	405
		SD	323	322	3	132	480
		Q1	44	11	0	0	35
		Q3	524	389	0	150	494
	Flip-Cover (n=3)	Mean	600	187	3	46	741
		Median	520	69	0	32	804
		SD	322	218	5	40	346
		Q1	326	54	0	14	368
		Q3	n.d.	n.d.	n.d.	n.d.	n.d.
	Case (n=73)	Mean	410	251	5	84	422
		Median	228	105	0	19	227

		**SD**	483	410	21	176	512
		**Q1**	88	44	0	0	60
		**Q3**	590	296	0	61	553
**Screen protector**	**No screen protector (n=31)**	**Mean**	376	252	4	57	370
		**Median**	222	100	0	10	227
		**SD**	459	436	12	110	451
		**Q1**	75	61	0	0	40
		**Q3**	383	289	0	44	481
	**Tempered glass (n=35)**	**Mean**	431	247	7	100	436
		**Median**	284	83	0	14	213
		**SD**	487	402	28	220	517
		**Q1**	88	42	0	0	76
		**Q3**	595	302	0	70	562
	**Plastic film (n=17)**	**Mean**	435	239	1	92	538
		**Median**	321	118	0	30	310
		**SD**	458	323	3	137	576
		**Q1**	94	18	0	10	151
		**Q3**	589	371	0	127	832
**Usual means of transport**	**Usually - Public transport (n=35)**	**Mean**	519	348	3	84	573
		**Median**	312	189	0	29	329
		**SD**	517	474	9	135	591
		**Q1**	132	43	0	0	132
		**Q3**	867	438	0	87	833
	**Usually - Private transport (n=48)**	**Mean**	332	174	6	82	329
		**Median**	214	82	0	14	168
		**SD**	412	313	24	191	405
		**Q1**	75	28	0	0	40
		**Q3**	434	161	0	50	469
**Means of transport in the day of sampling**	**Today - Public transport (n=28)**	**Mean**	577	392	3	98	600
		**Median**	397	212	0	31	339
		**SD**	559	520	10	155	620
		**Q1**	141	58	0	0	107
		**Q3**	853	453	0	133	1015
	**Today - Private transport (n=53)**	**Mean**	329	174	5	75	347
		**Median**	200	81	0	14	170
		**SD**	397	299	23	180	420
		**Q1**	72	25	0	0	44
		**Q3**	409	155	0	44	500
	**Today - Both public and private transport (n=2)**	**Mean**	265	167	4	55	316
		**Median**	265	167	4	55	316
		**SD**	67	191	6	16	126
		**Q1**	217	32	0	43	227
		**Q3**	n.d.	n.d.	n.d.	n.d.	n.d.
**Use with gloves**	**No (n=64)**	**Mean**	451	264	5	94	449
		**Median**	287	83	0	29	296
		**SD**	501	442	22	179	484
		**Q1**	102	28	0	0	95
		**Q3**	618	301	0	82	603
	**Yes**	**Mean**	241	233	2	23	428

	**(n=10)**	**Median**	143	186	0	10	153
		**SD**	234	169	4	43	654
		**Q1**	88	75	0	0	52
		**Q3**	456	391	2	22	630
	**Yes, changing gloves (n=9)**	**Mean**	314	140	5	68	316
		**Median**	144	105	0	0	79
		**SD**	359	136	11	178	504
		**Q1**	61	45	0	0	26
		**Q3**	531	236	5	30	504
**Health status**	**Ill (n=14)**	**Mean**	520	347	15	168	521
		**Median**	286	82	0	37	258
		**SD**	617	538	43	302	523
		**Q1**	104	37	0	7	166
		**Q3**	871	524	9	195	929
	**Healthy (n=68)**	**Mean**	389	227	3	65	414
		**Median**	228	105	0	14	238
		**SD**	431	362	9	123	501
		**Q1**	83	37	0	0	49
		**Q3**	556	300	0	51	536
**European Head SAR (Mean = 0.86 W/kg)**	**Above average (n=50)**	**Mean**	507	301	6	101	515
		**Median**	294	103	0	26	302
		**SD**	531	487	24	199	548
		**Q1**	126	44	0	7	78
		**Q3**	791	341	0	74	811
	**Below average (n=33)**	**Mean**	266	165	2	54	306
		**Median**	170	83	0	10	170
		**SD**	296	164	7	104	403
		**Q1**	76	24	0	0	31
		**Q3**	361	296	0	45	458

Radar Plots showing the bacterial charges across the 15 selected user's demographics and devices characteristics

Figure S1: Radar plot for Gender

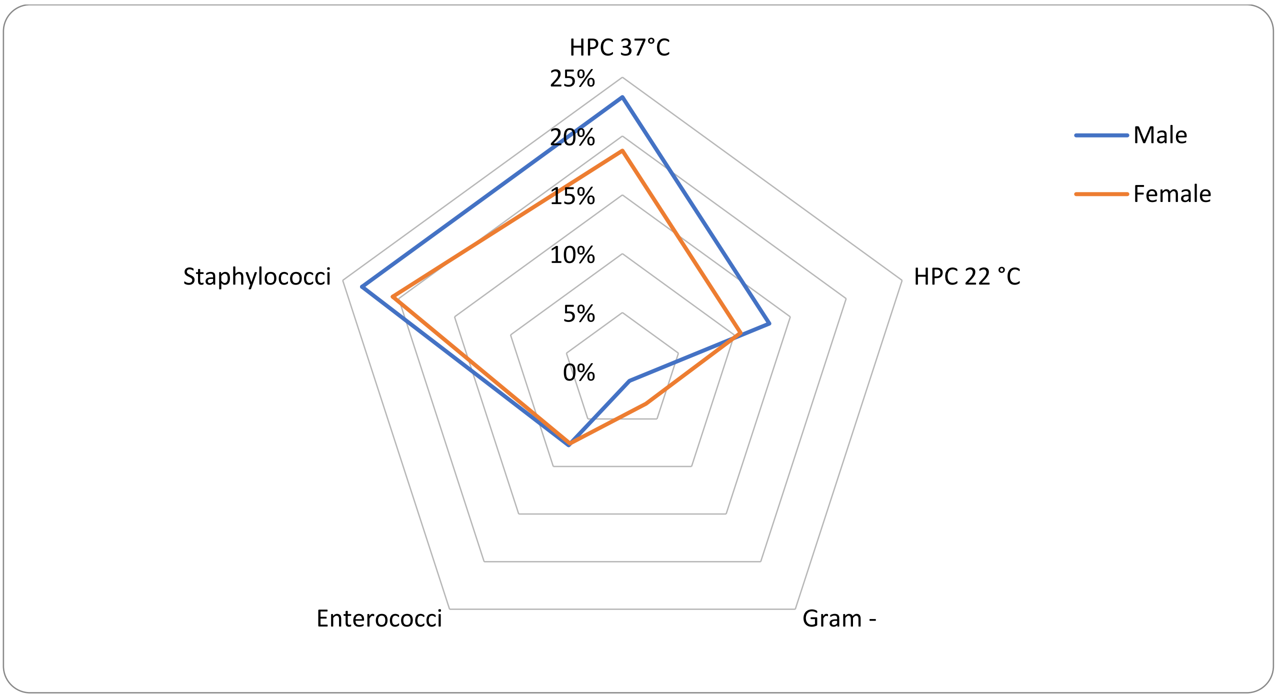

Figure S2: Radar plot for User's Age

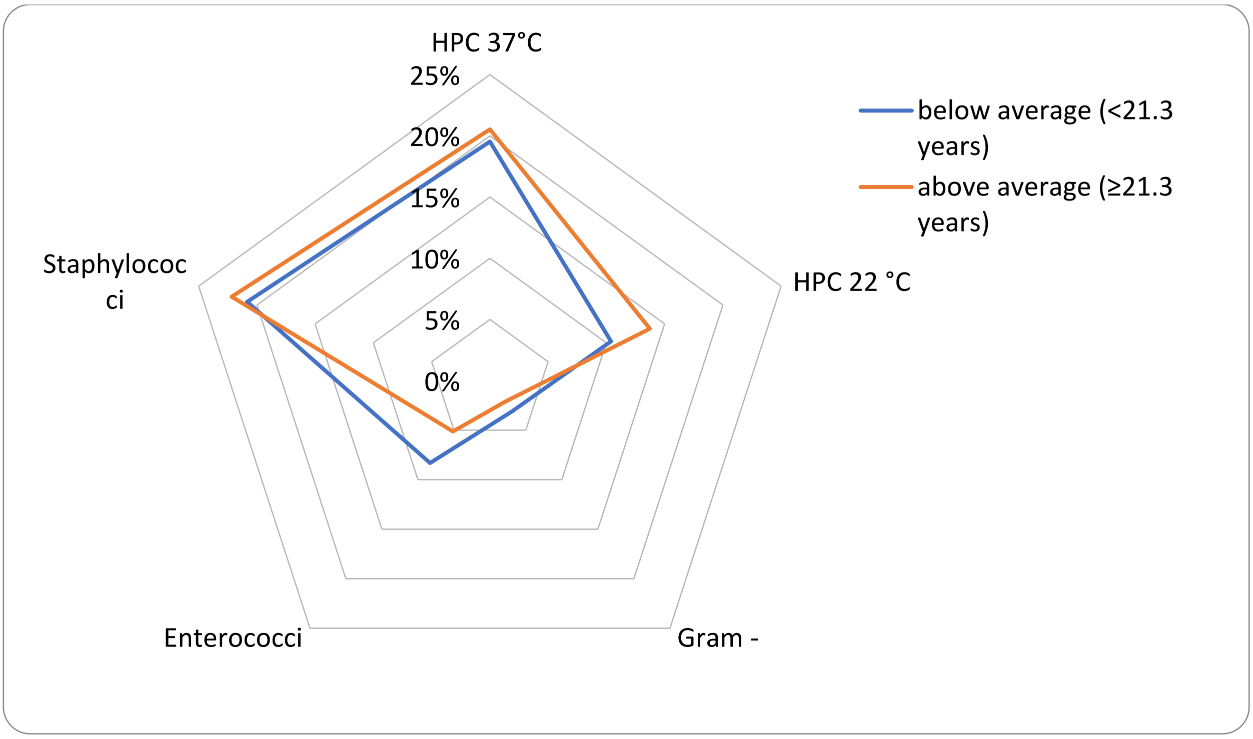

Figure S3: Radar plot for Place of training

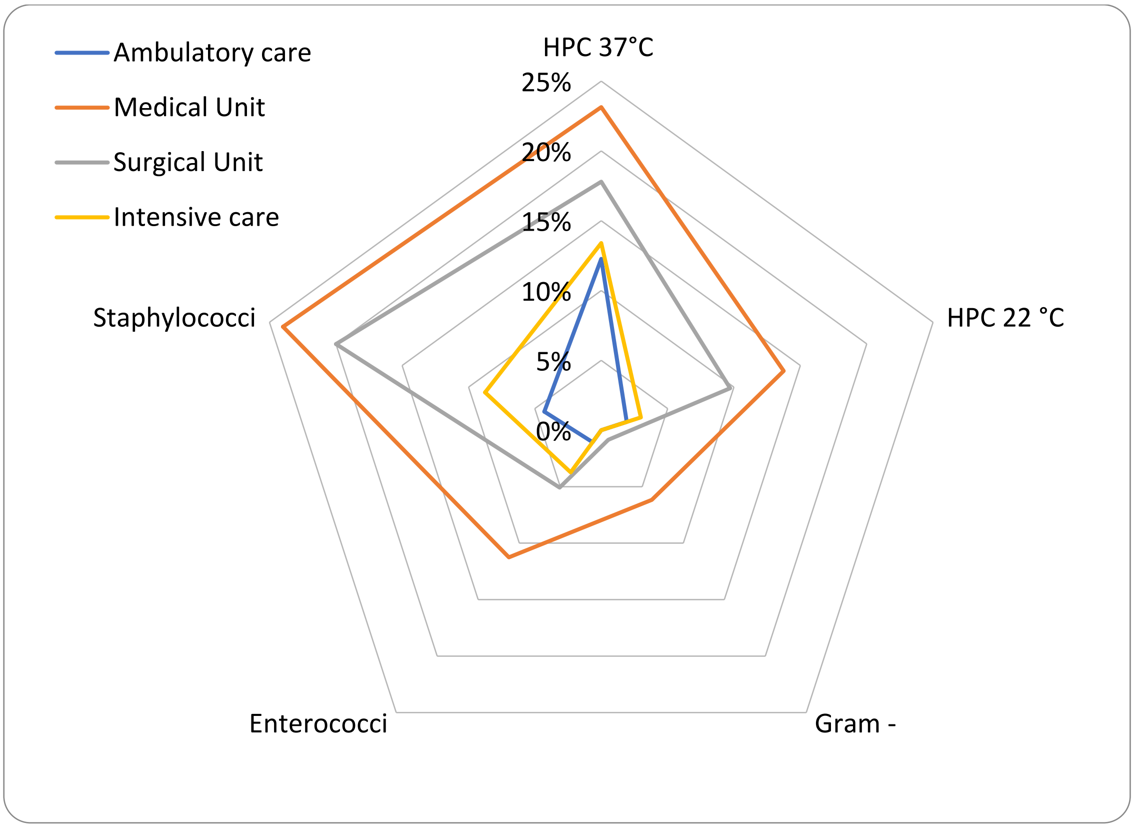

Figure S4: Radar plot for Training frequency

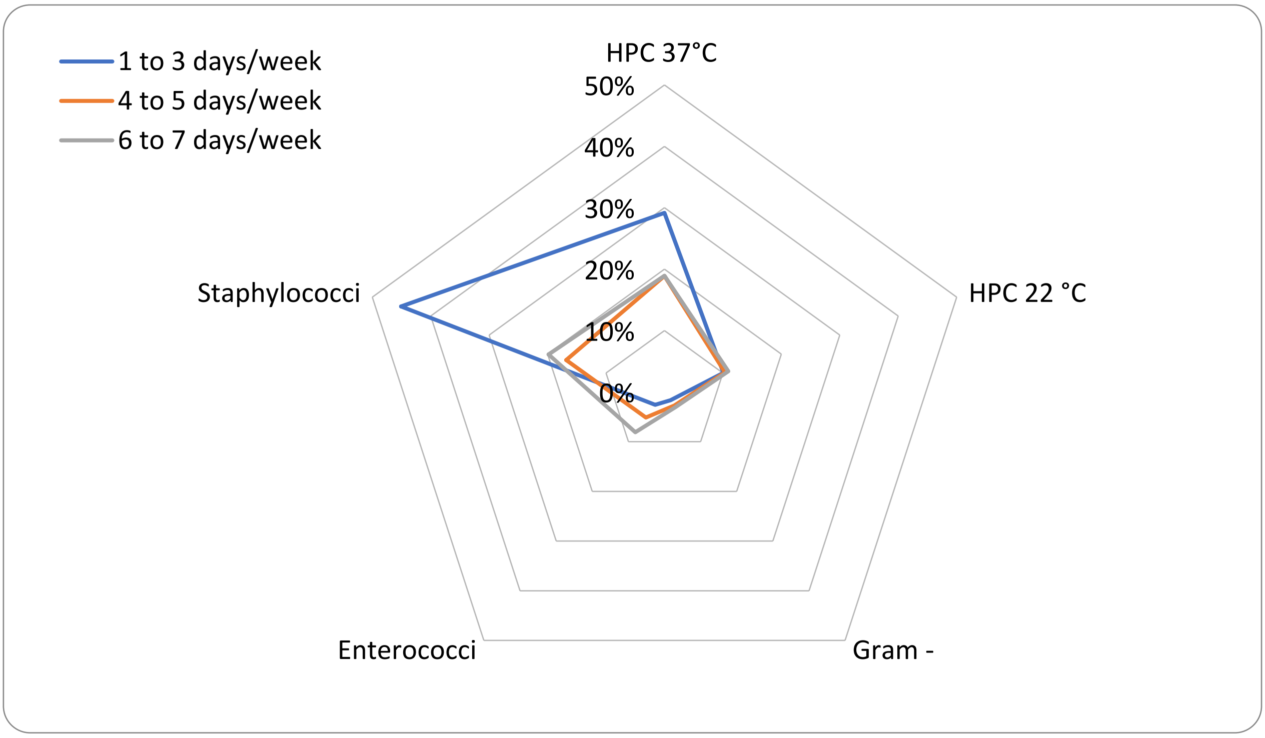

Figure S5: Radar plot for European Head SAR

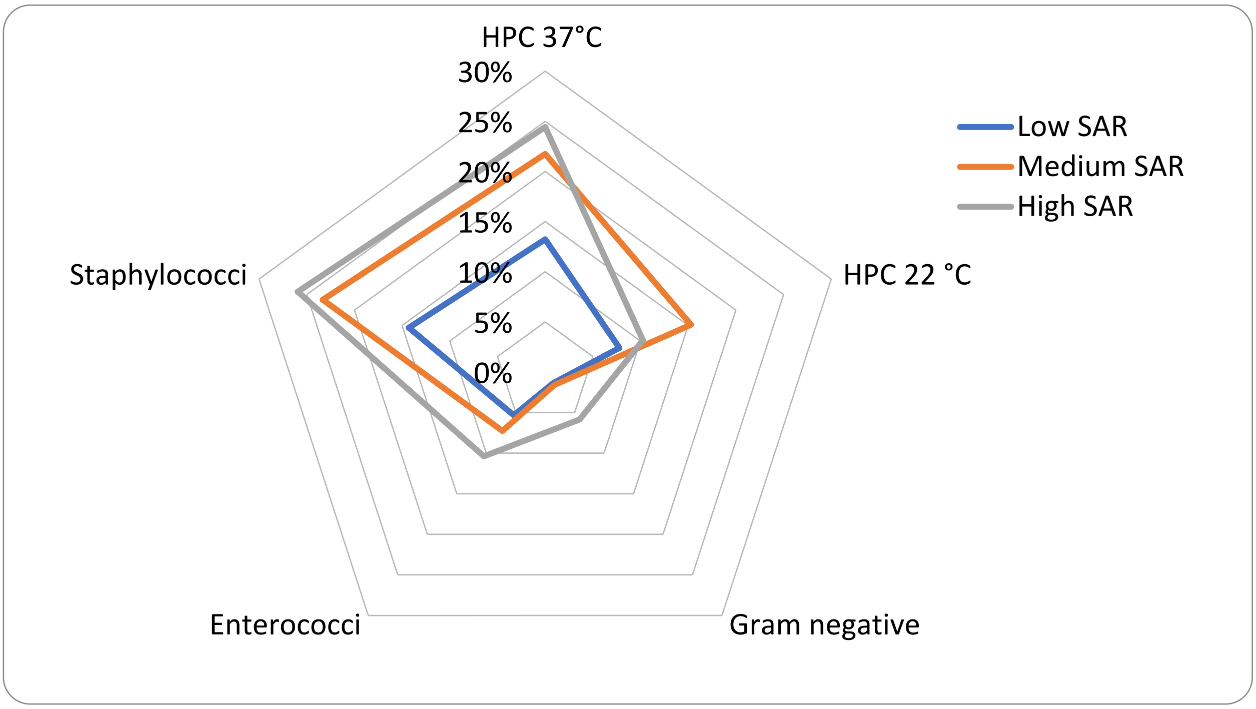

Figure S6: Radar plot for Smartphone age

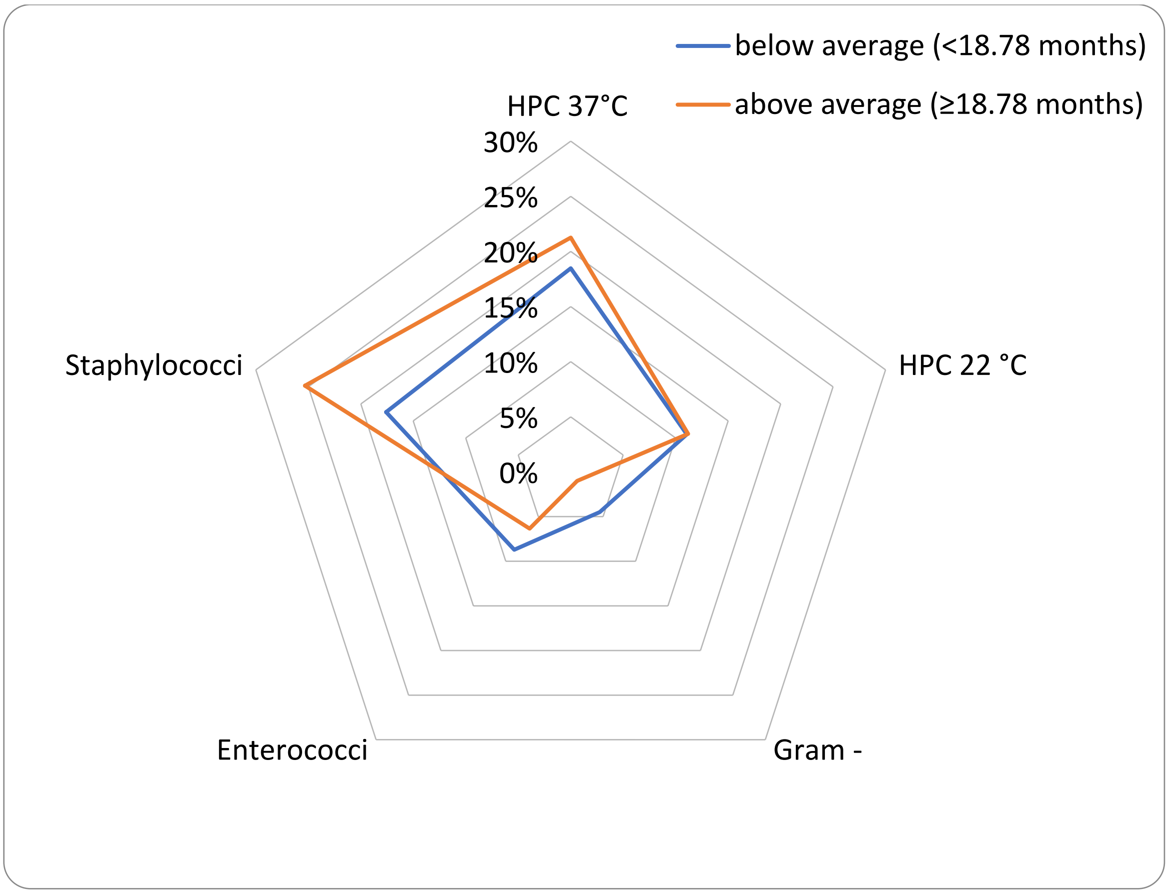

Figure S7: Radar plot for Cover type

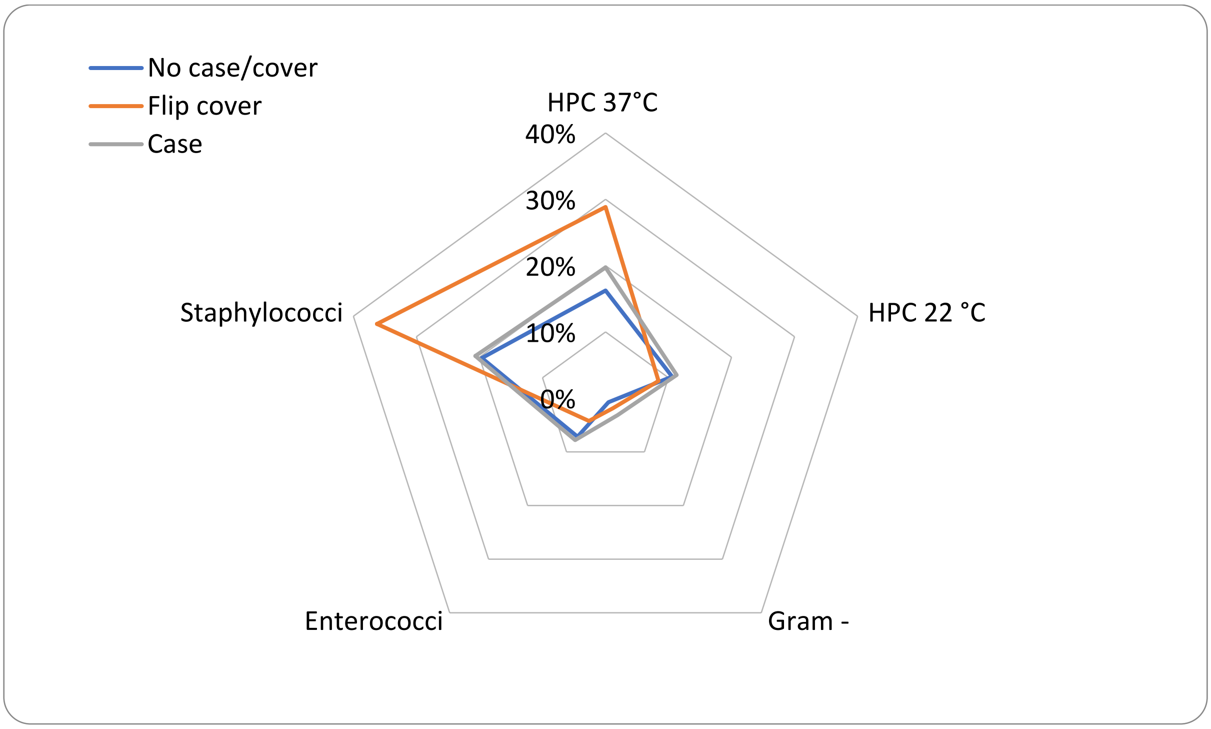

Figure S8: Radar plot for Screen protector

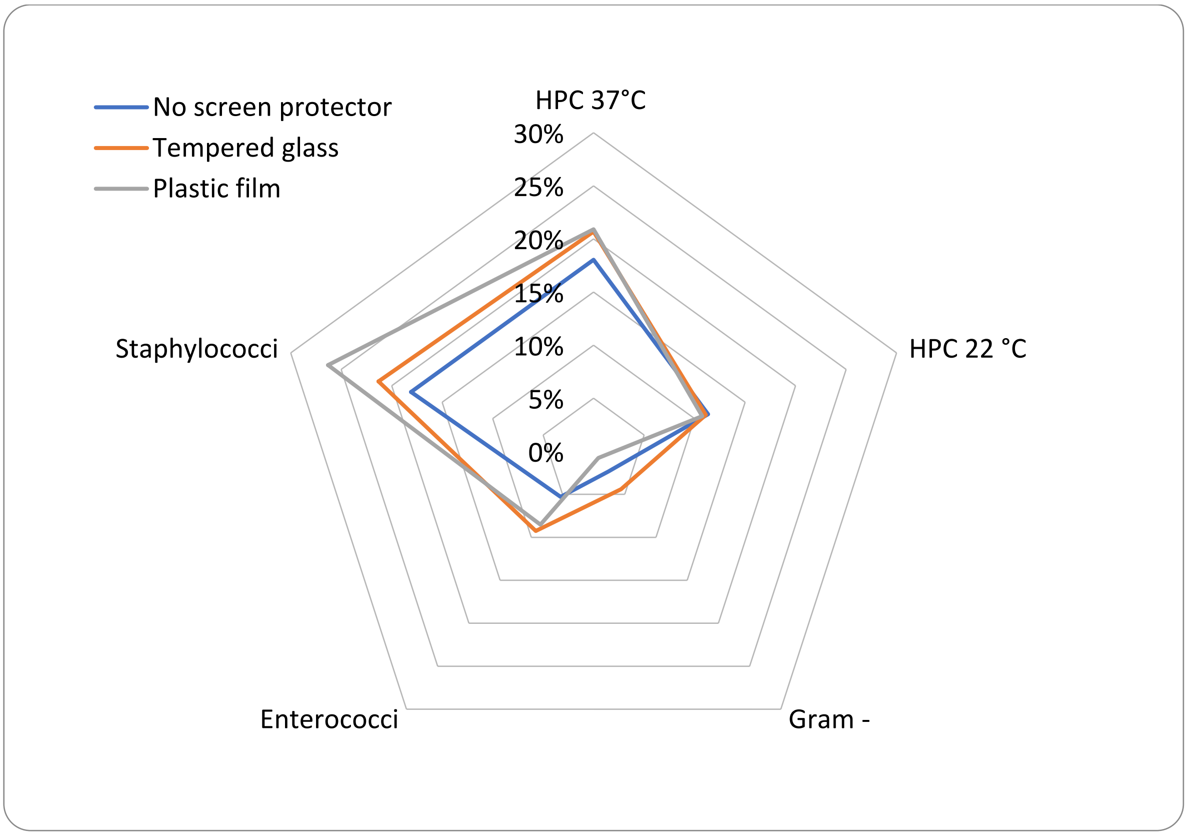

Figure S9: Radar plot for Health status

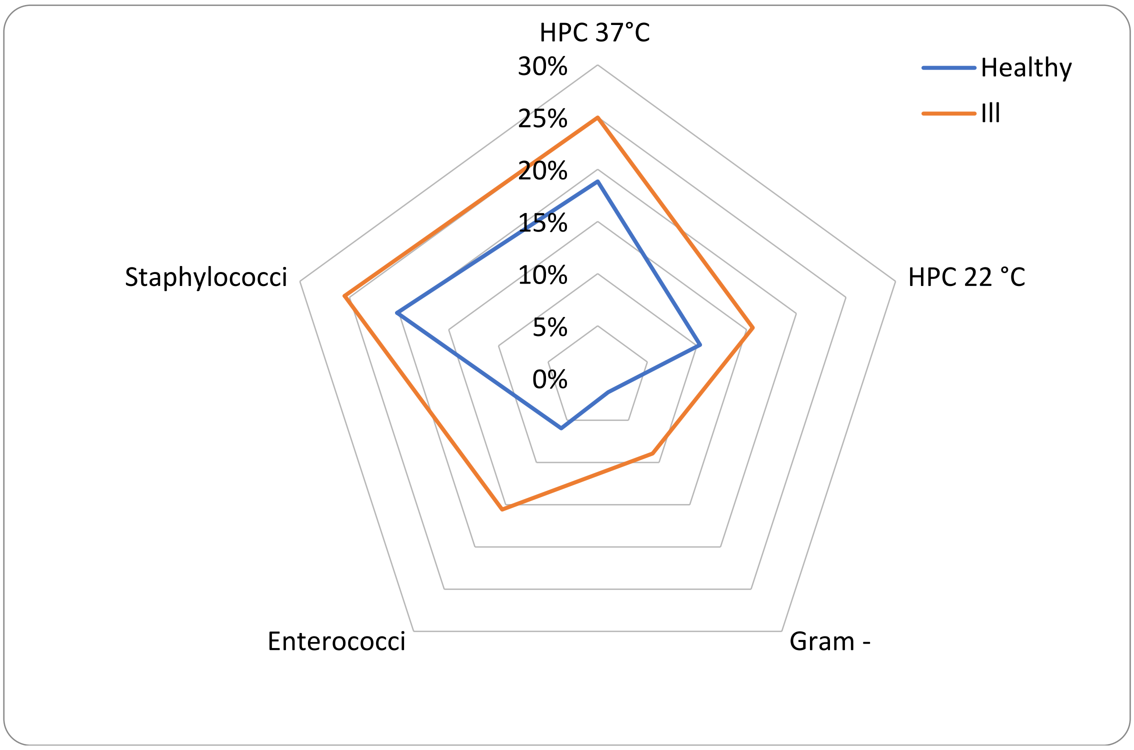

Figure S10: Radar plot for Cleaning frequency

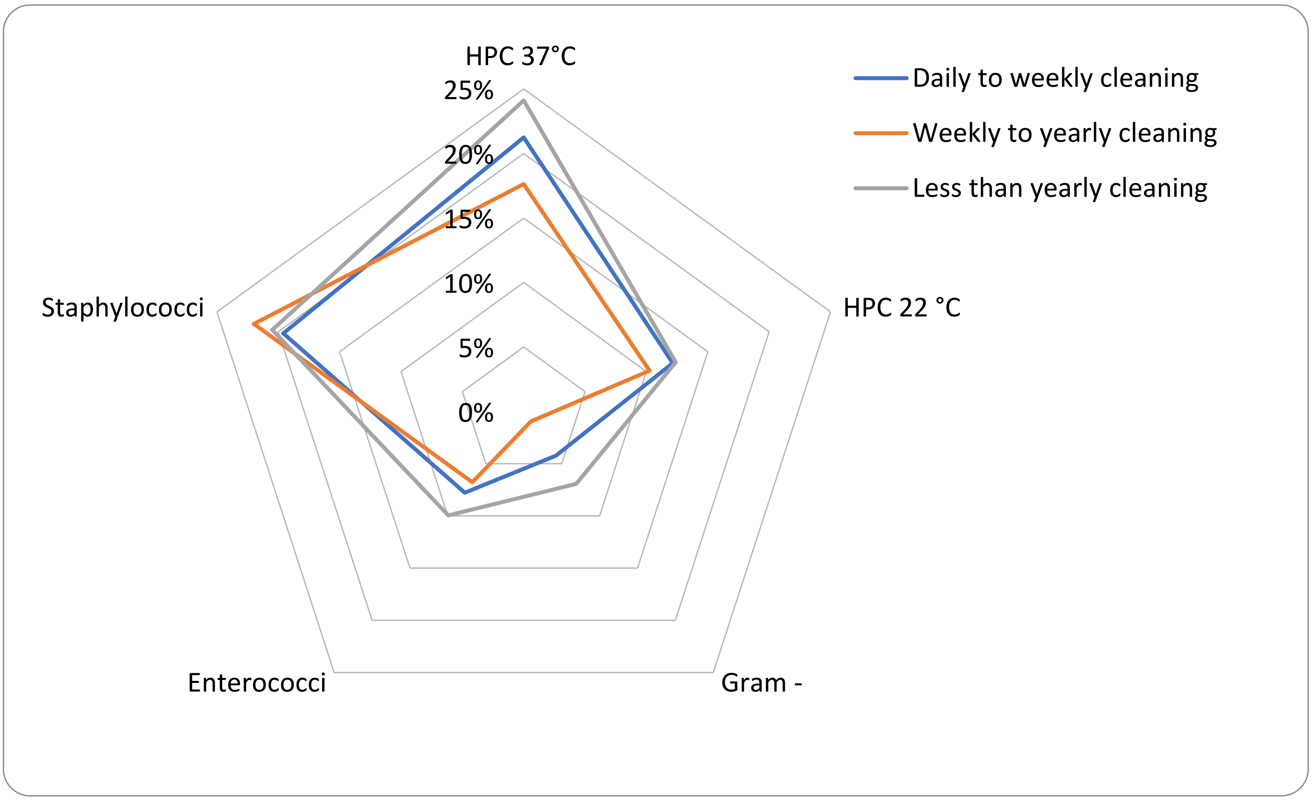

Figure S11: Radar plot for Cleaning method

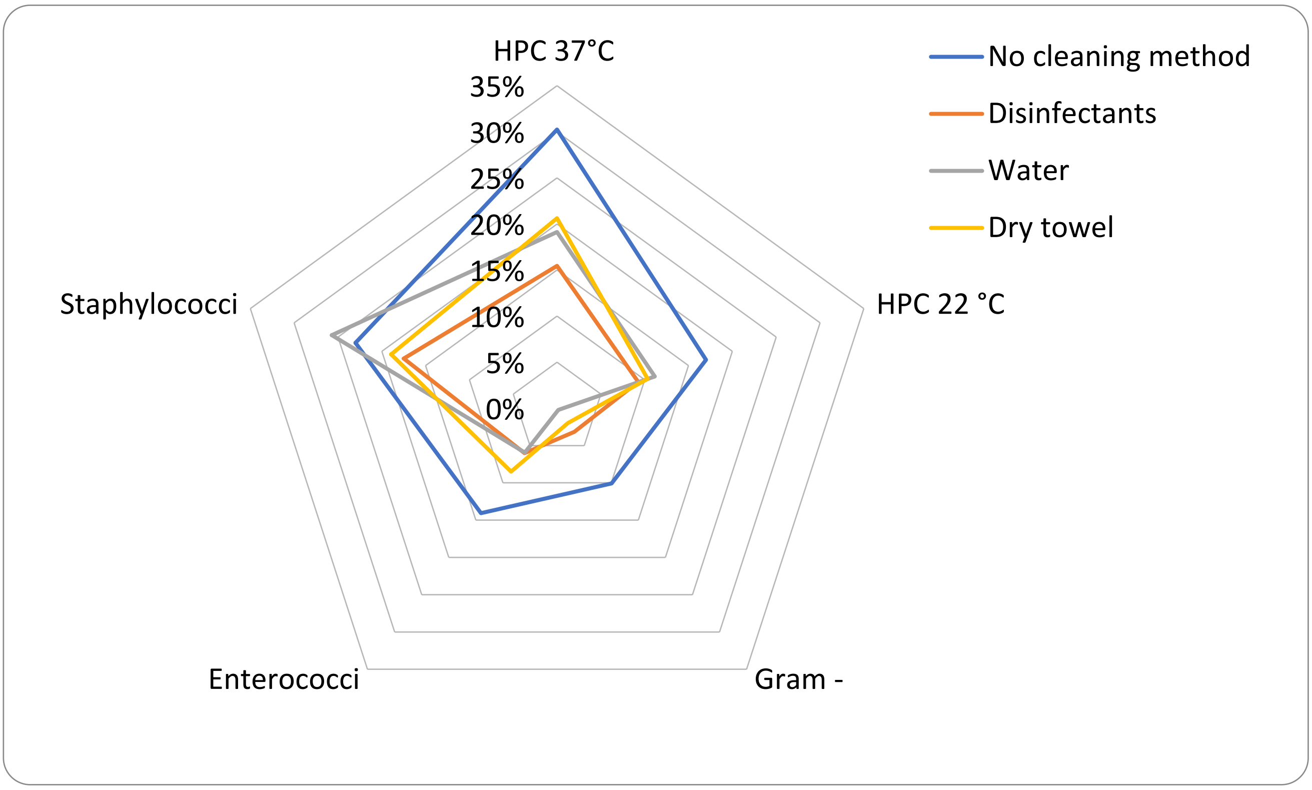

Figure S12: Radar plot for Last cleaning

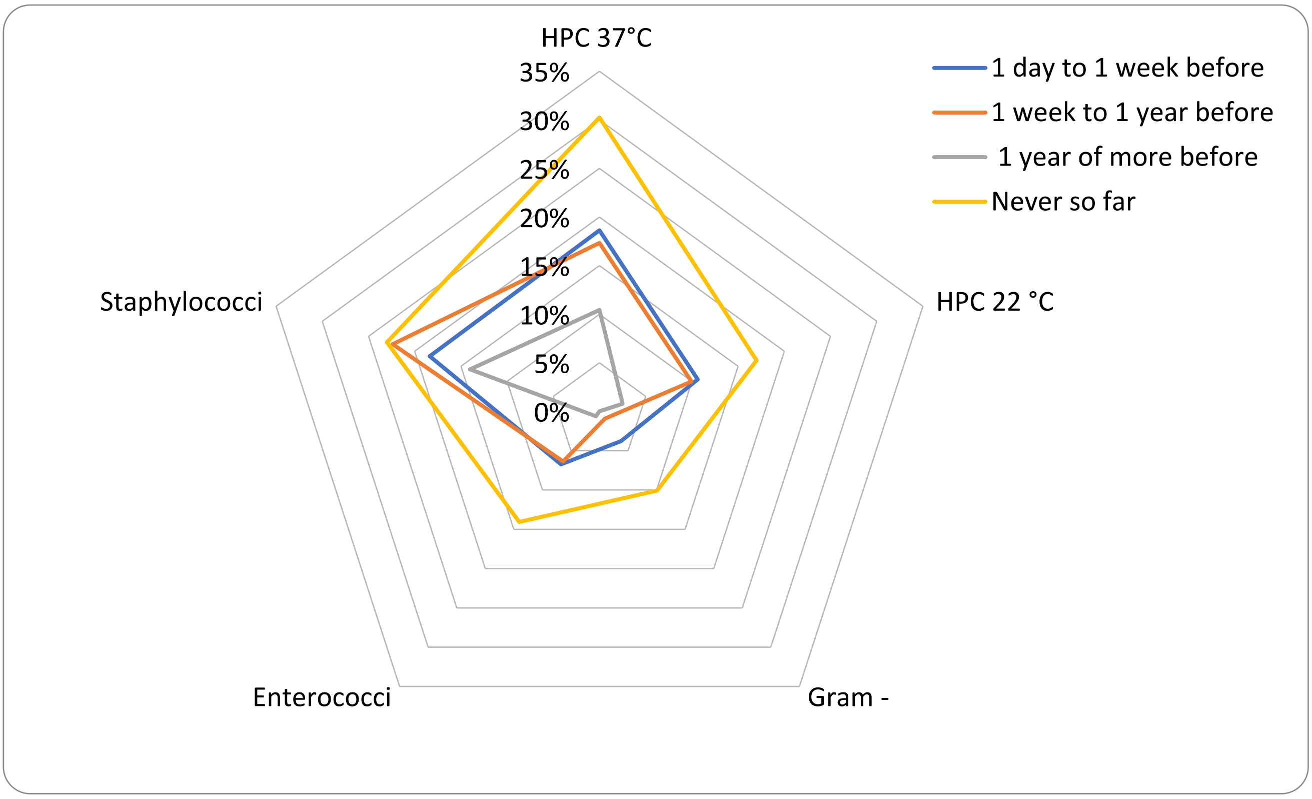

Figure S13: Radar plot for Use with gloves

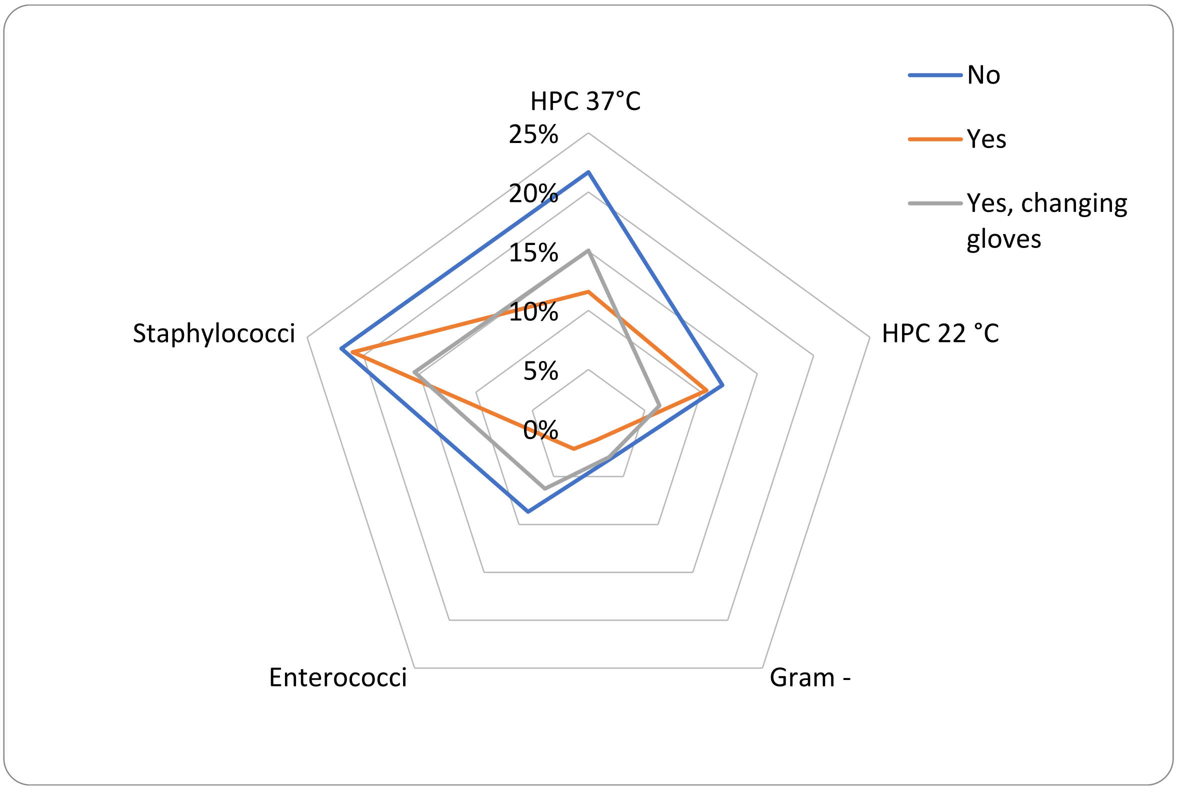

Figure S14: Radar plot for Usual means of transport

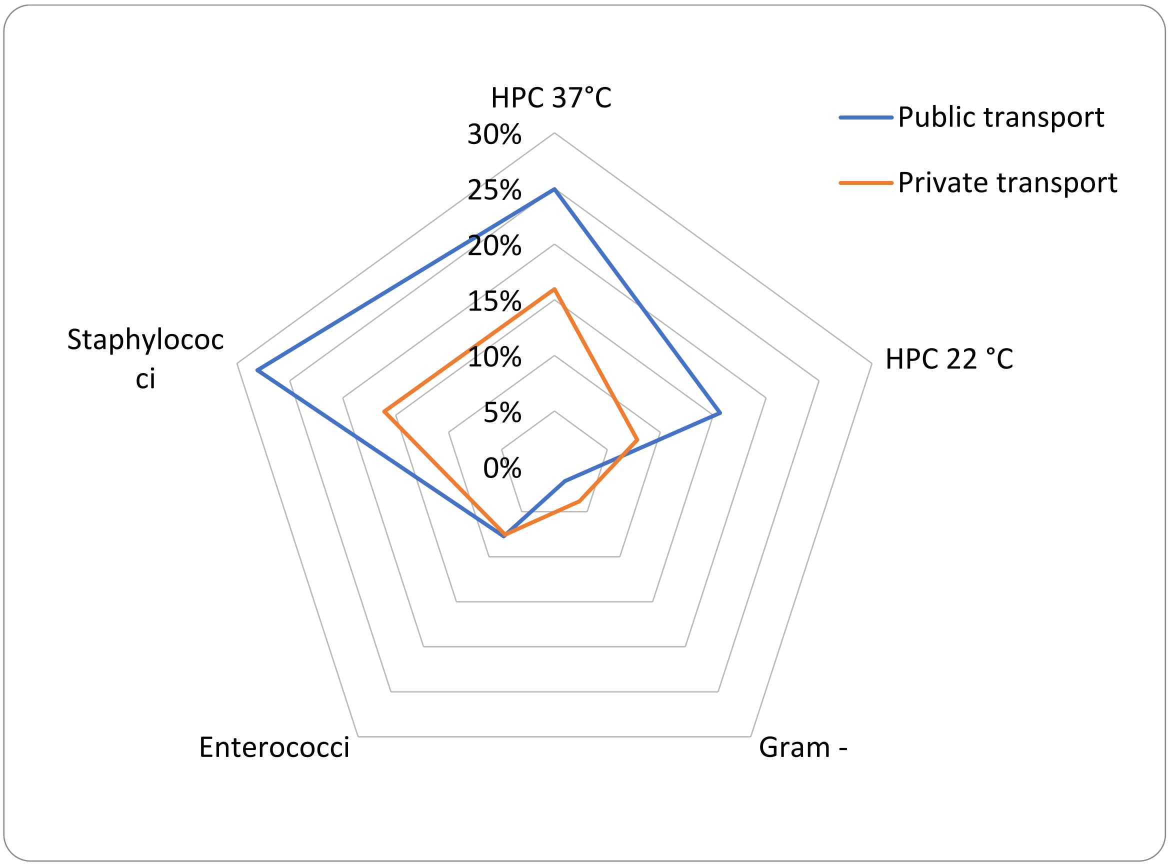

Figure S15: Radar plot for Means of transport in the day of sampling

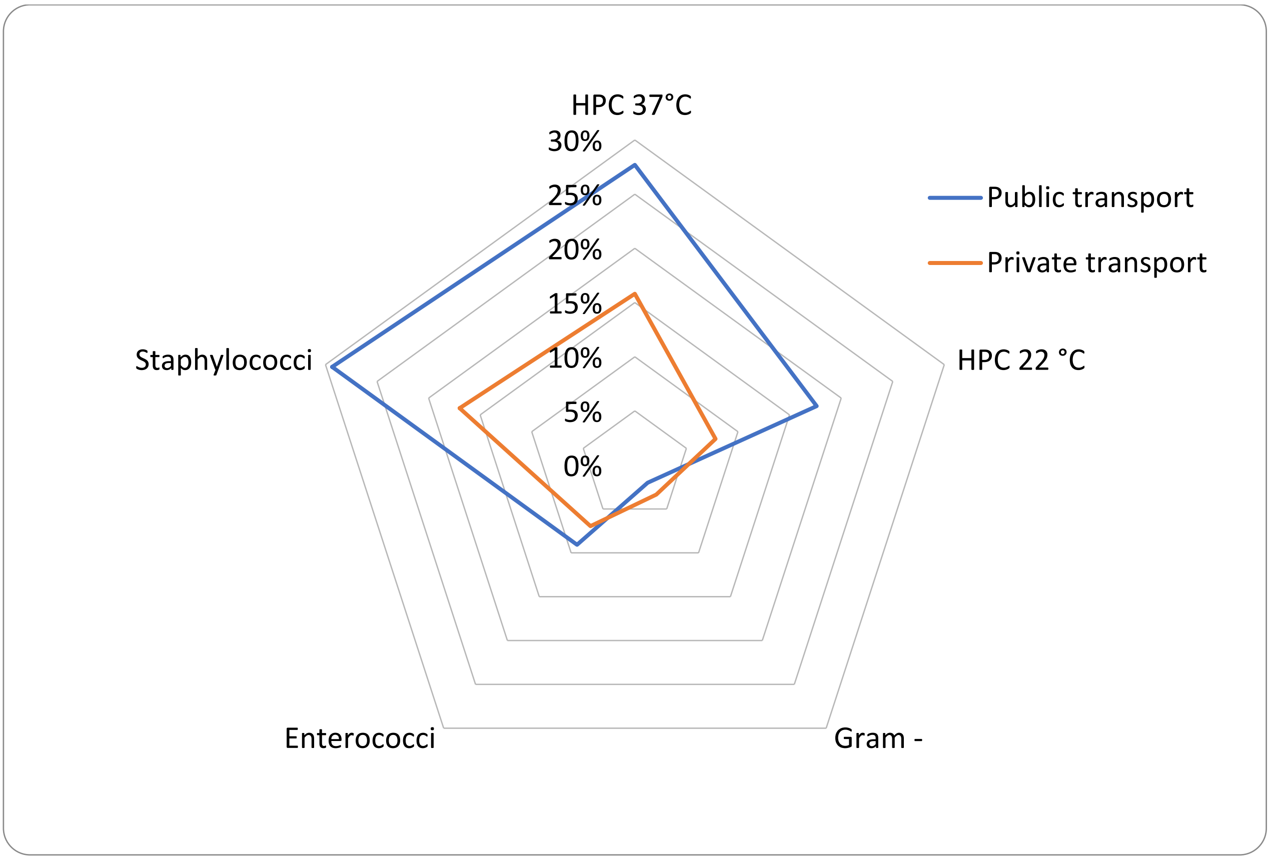

Scatter Plots comparing mean HPCs 37 °C and HPCs 22 °C across the selected variables

Figure S16: HPCs 37 °C and HPCs 22 °C based on Age

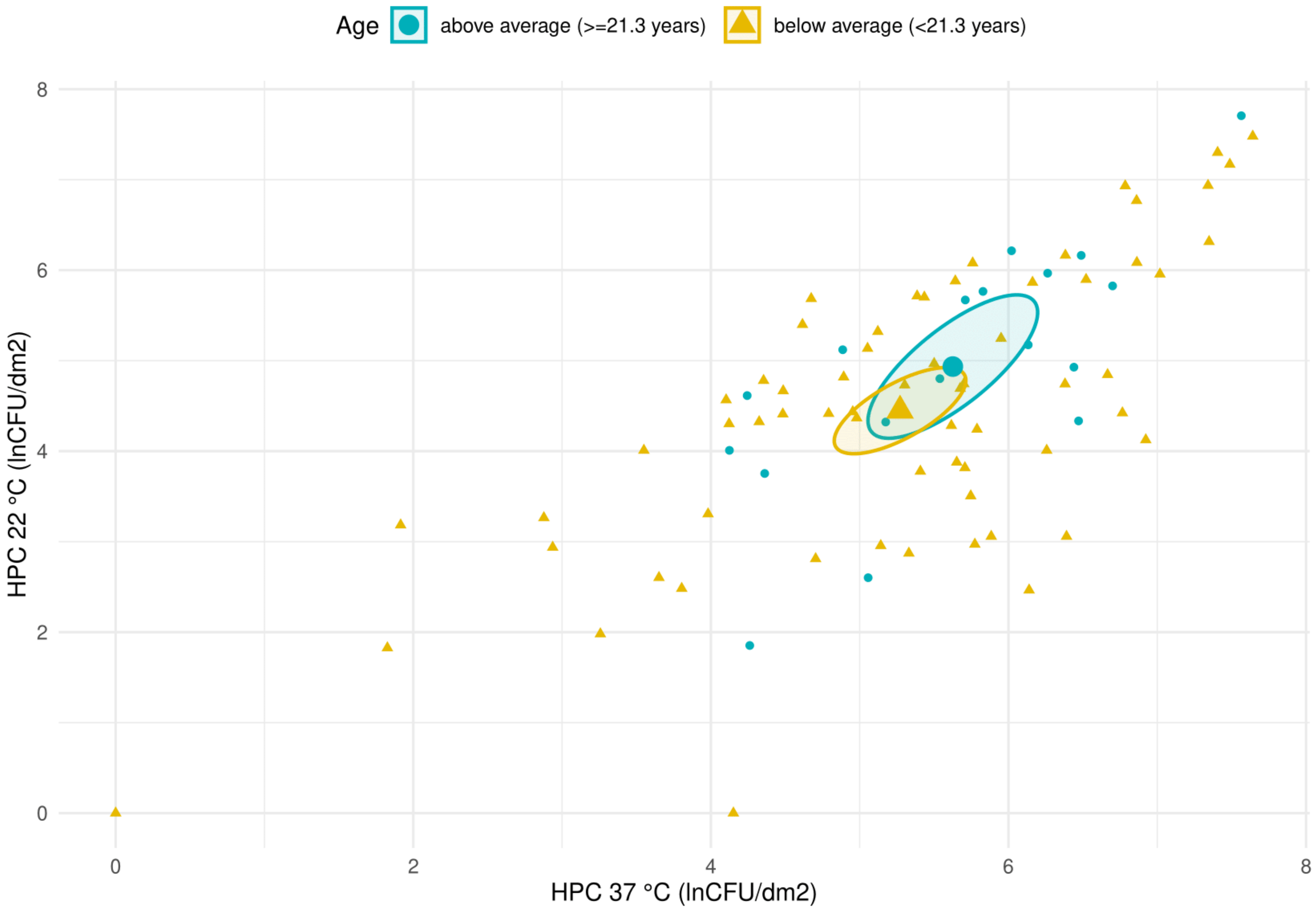

Figure S17: HPCs 37 °C and HPCs 22 °C based on Cleaning frequency

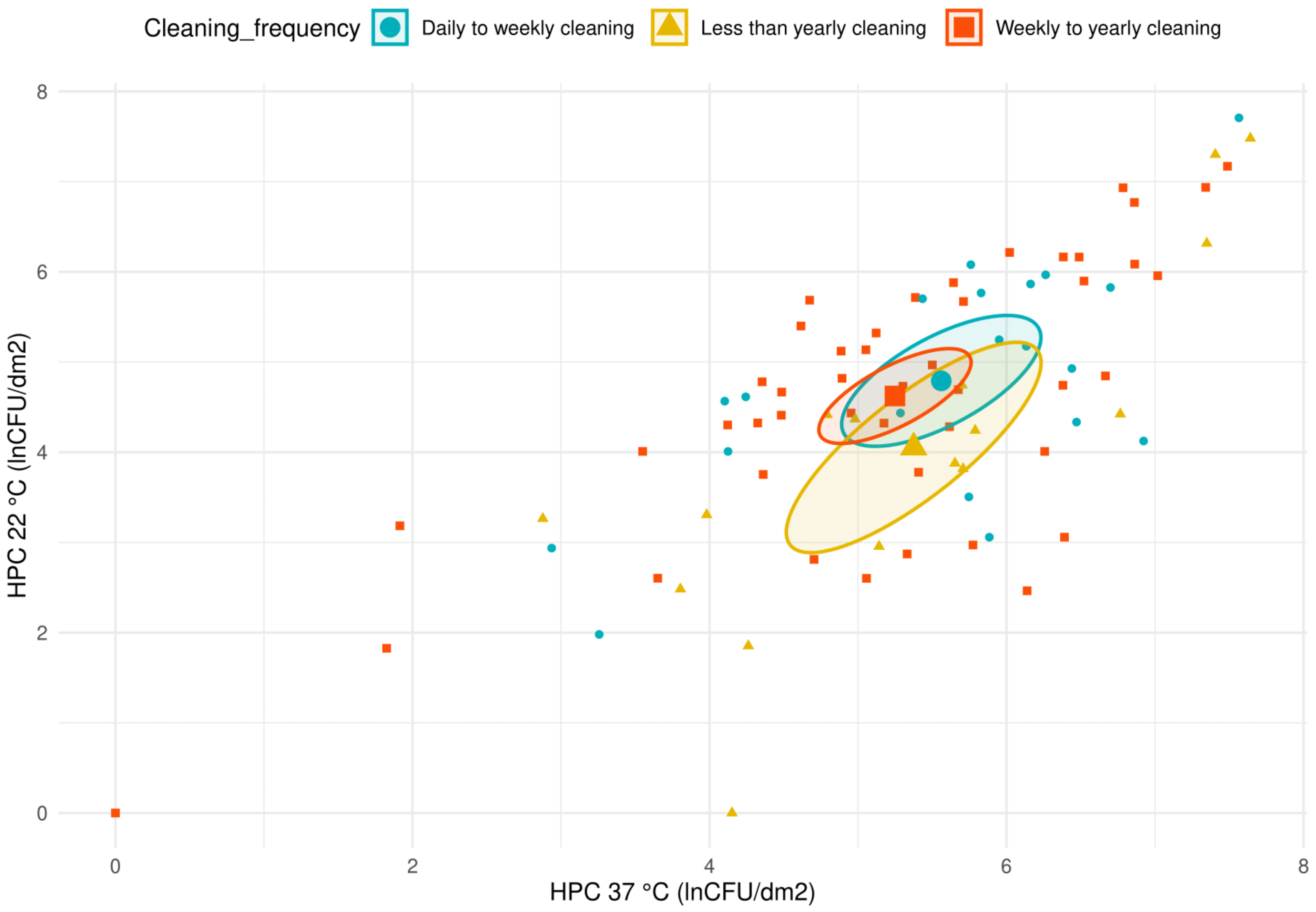

Figure S18: HPCs 37 °C and HPCs 22 °C based on Cleaning method

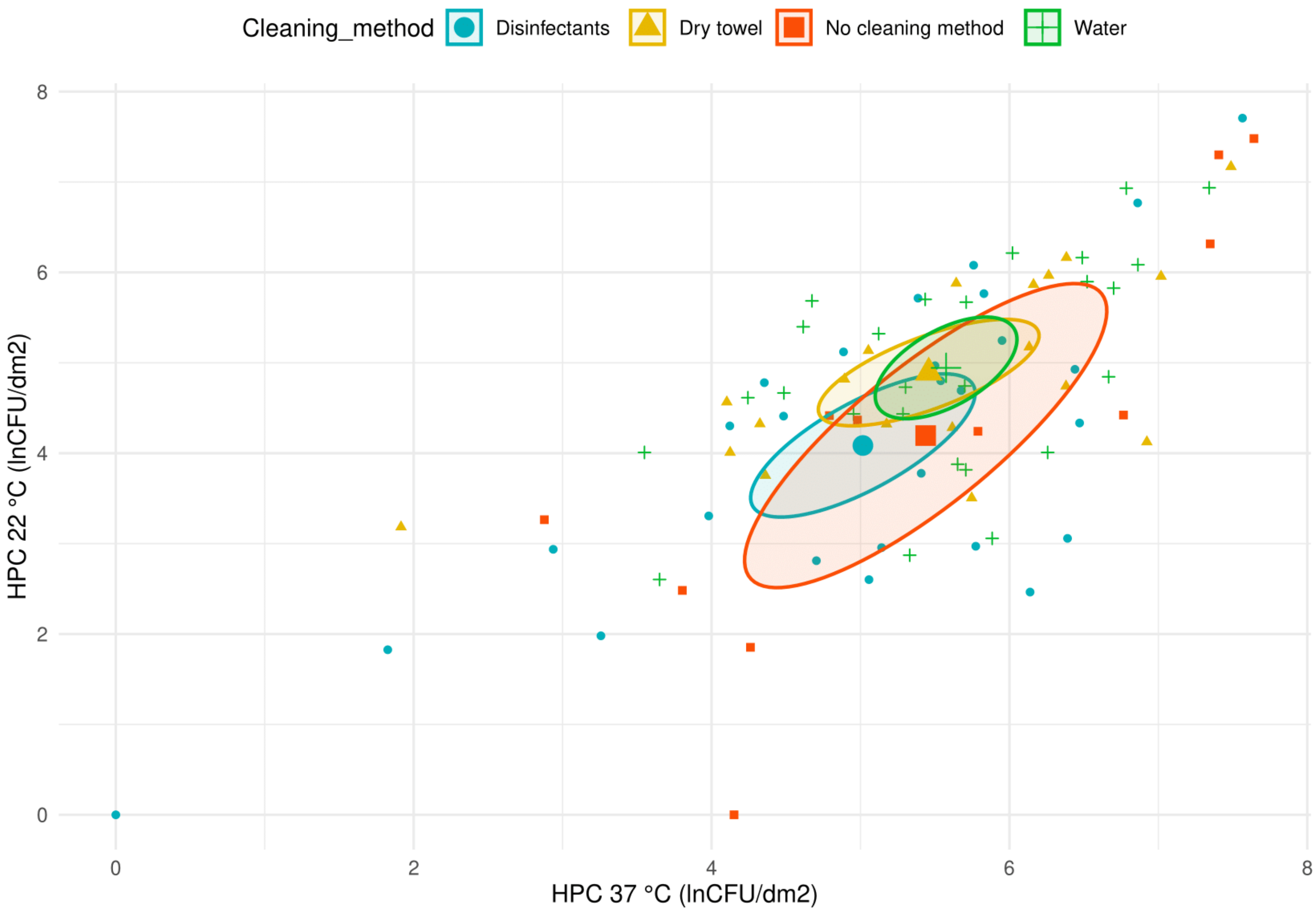

Figure S19: HPCs 37 °C and HPCs 22 °C based on Cover type

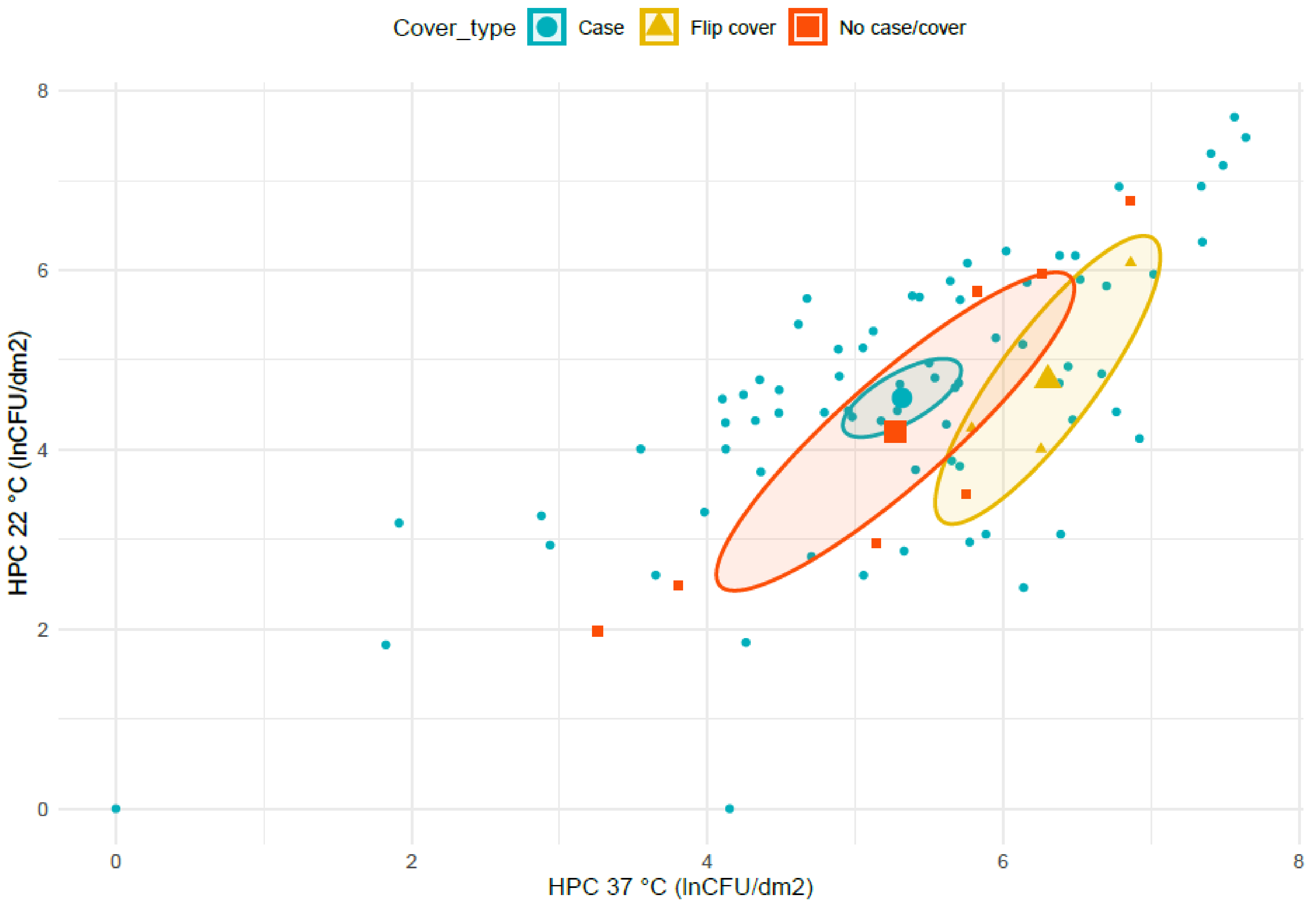

Figure S20: HPCs 37 °C and HPCs 22 °C based on European Head SAR (W/kg)

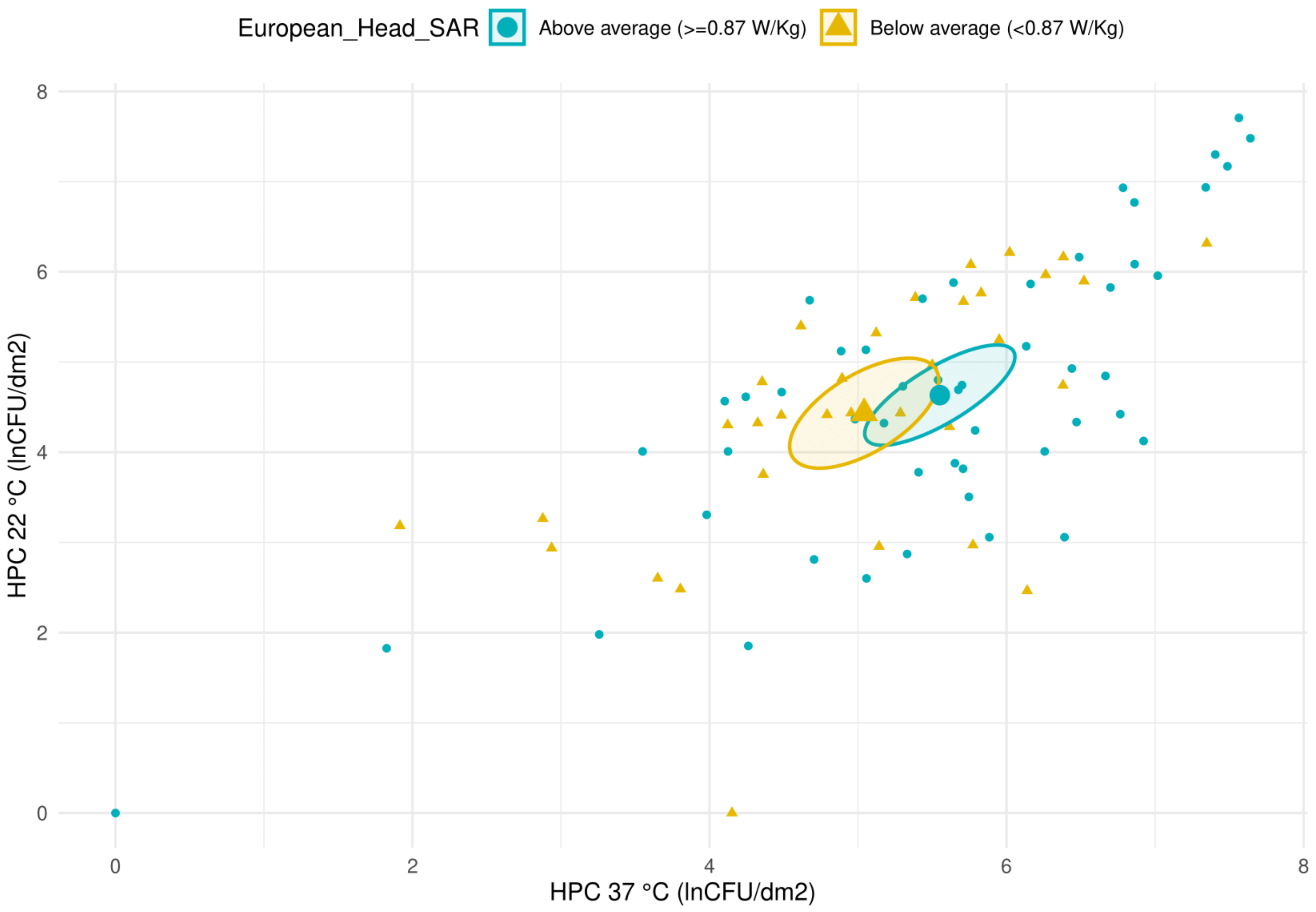

Figure S21: HPCs 37 °C and HPCs 22 °C based on Gender

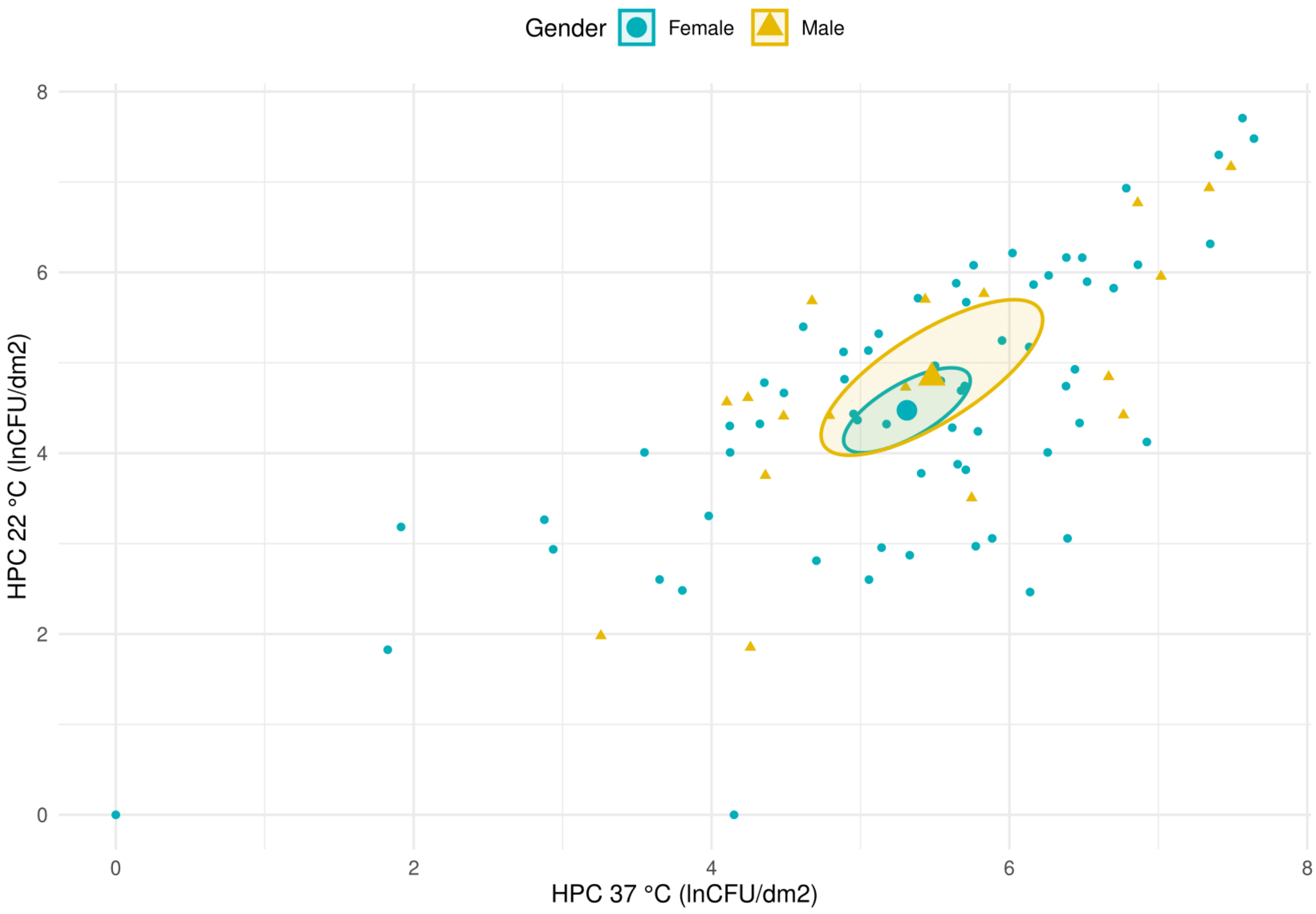

Figure S22: HPCs 37 °C and HPCs 22 °C based on Health Status

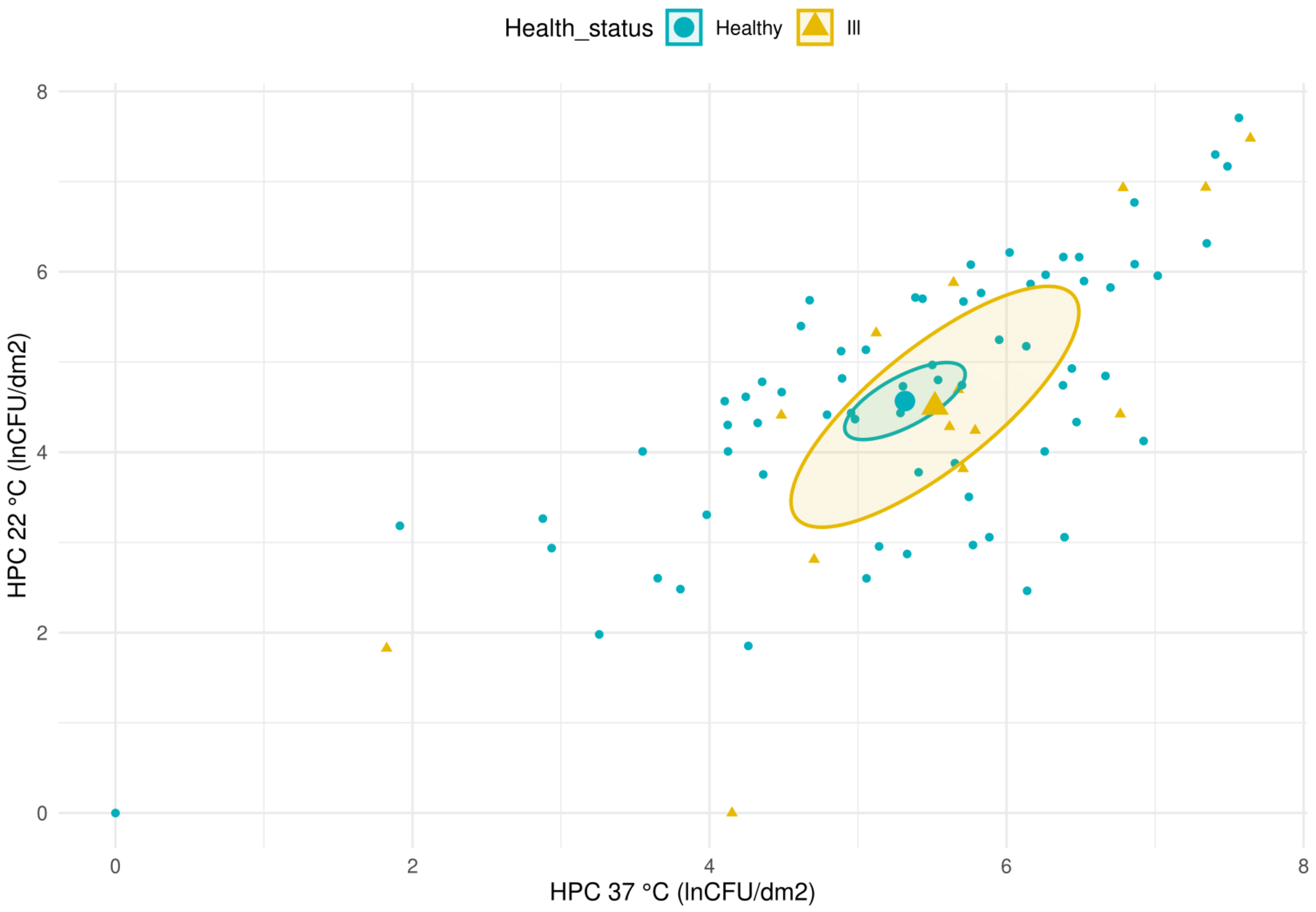

Figure S23: HPCs 37 °C and HPCs 22 °C based on Last Cleaning

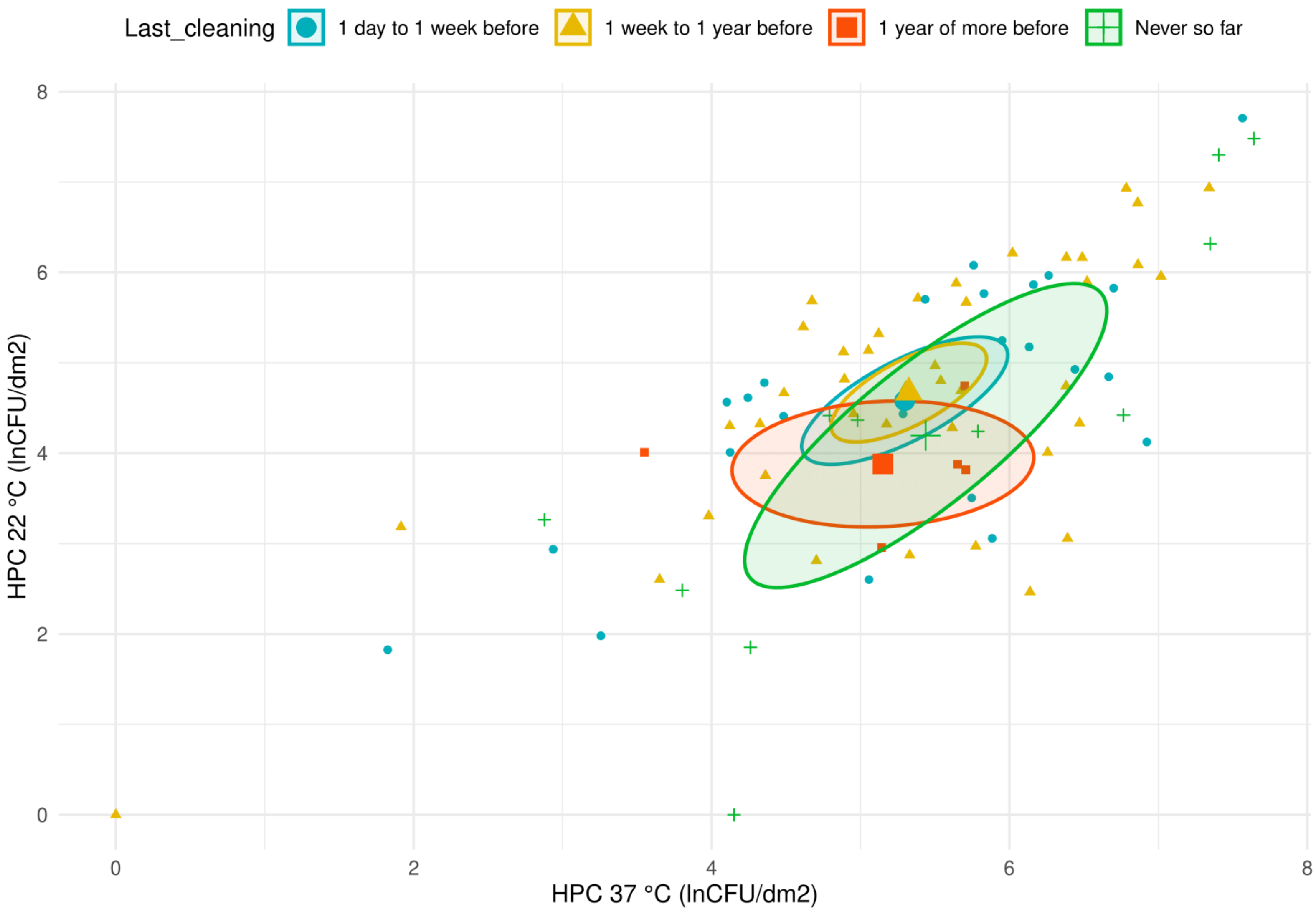

Figure S24: HPCs 37 °C and HPCs 22 °C based on Means of transport in the day of sampling

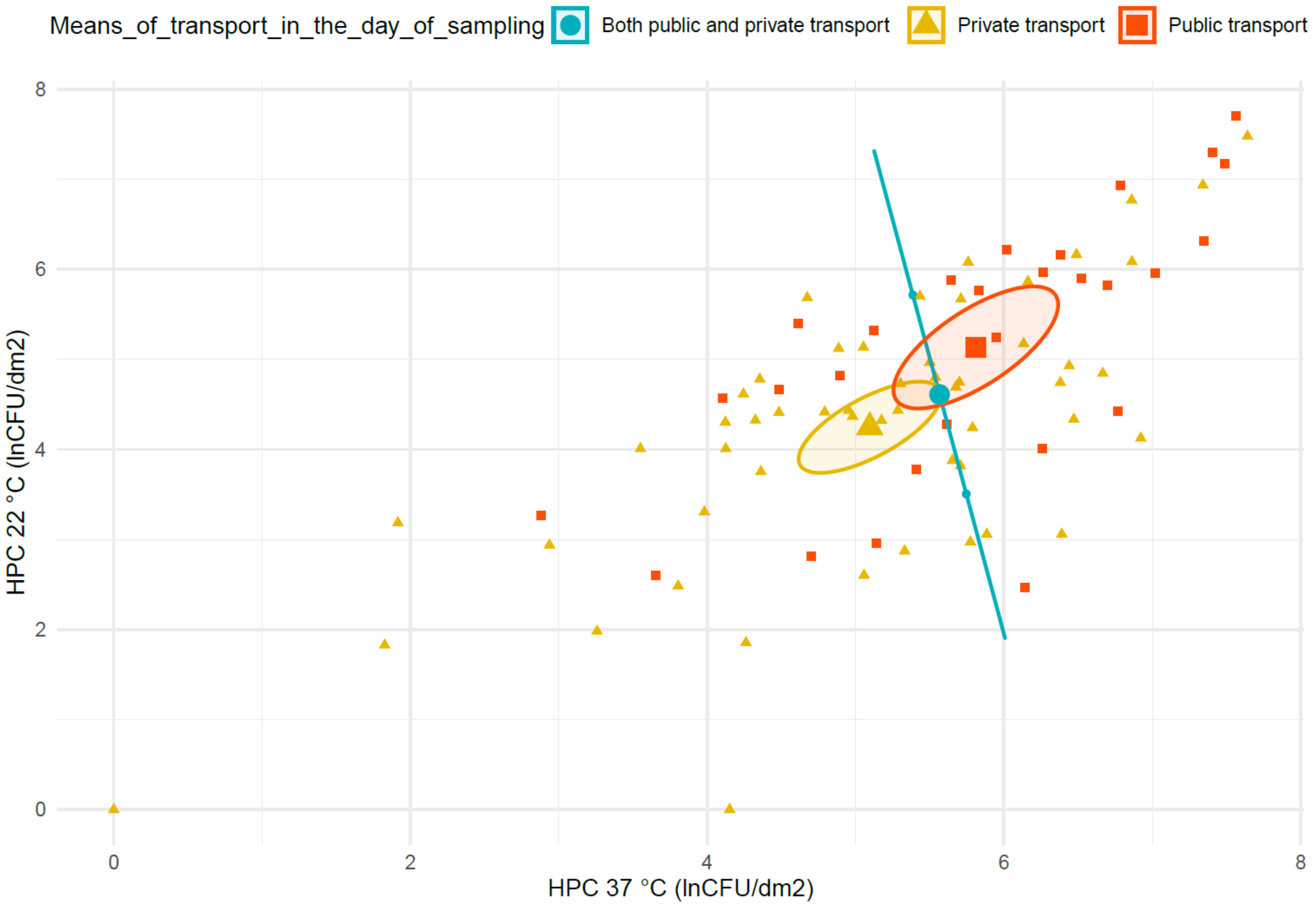

Figure S25: HPCs 37 °C and HPCs 22 °C based on Place of training

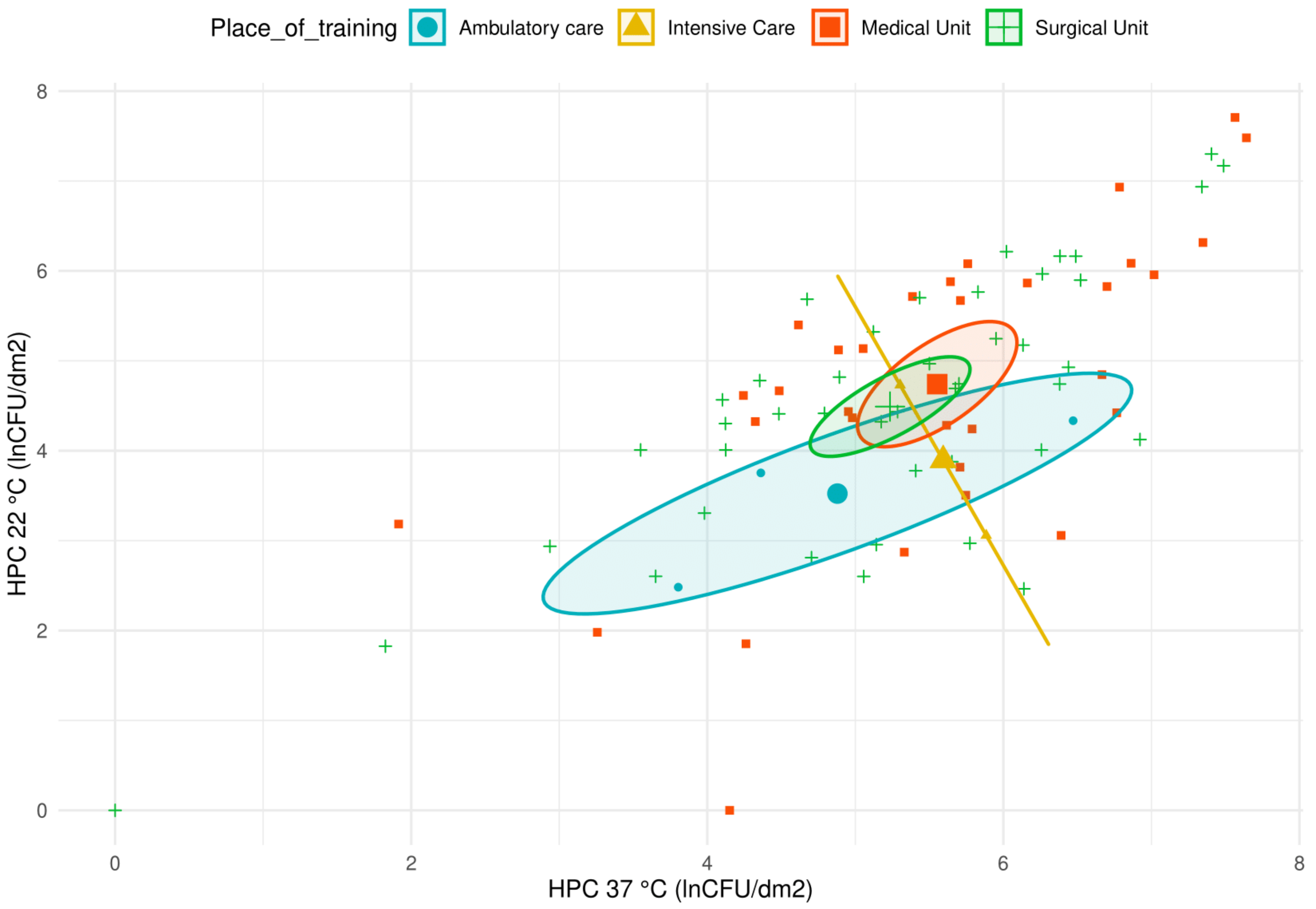

Figure S26: HPCs 37 °C and HPCs 22 °C based on Screen protector

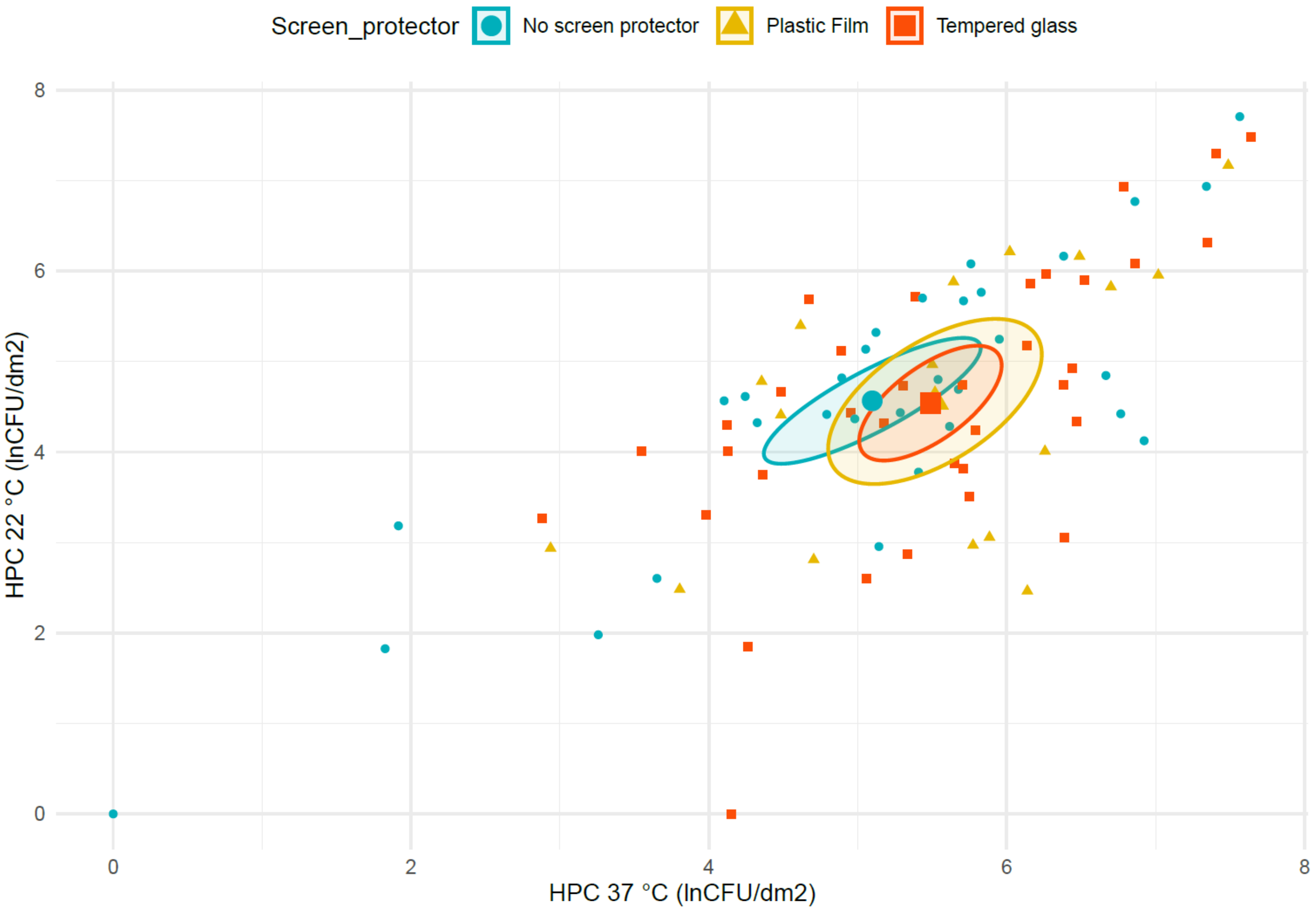

Figure S27: HPCs 37 °C and HPCs 22 °C based on Smartphone age

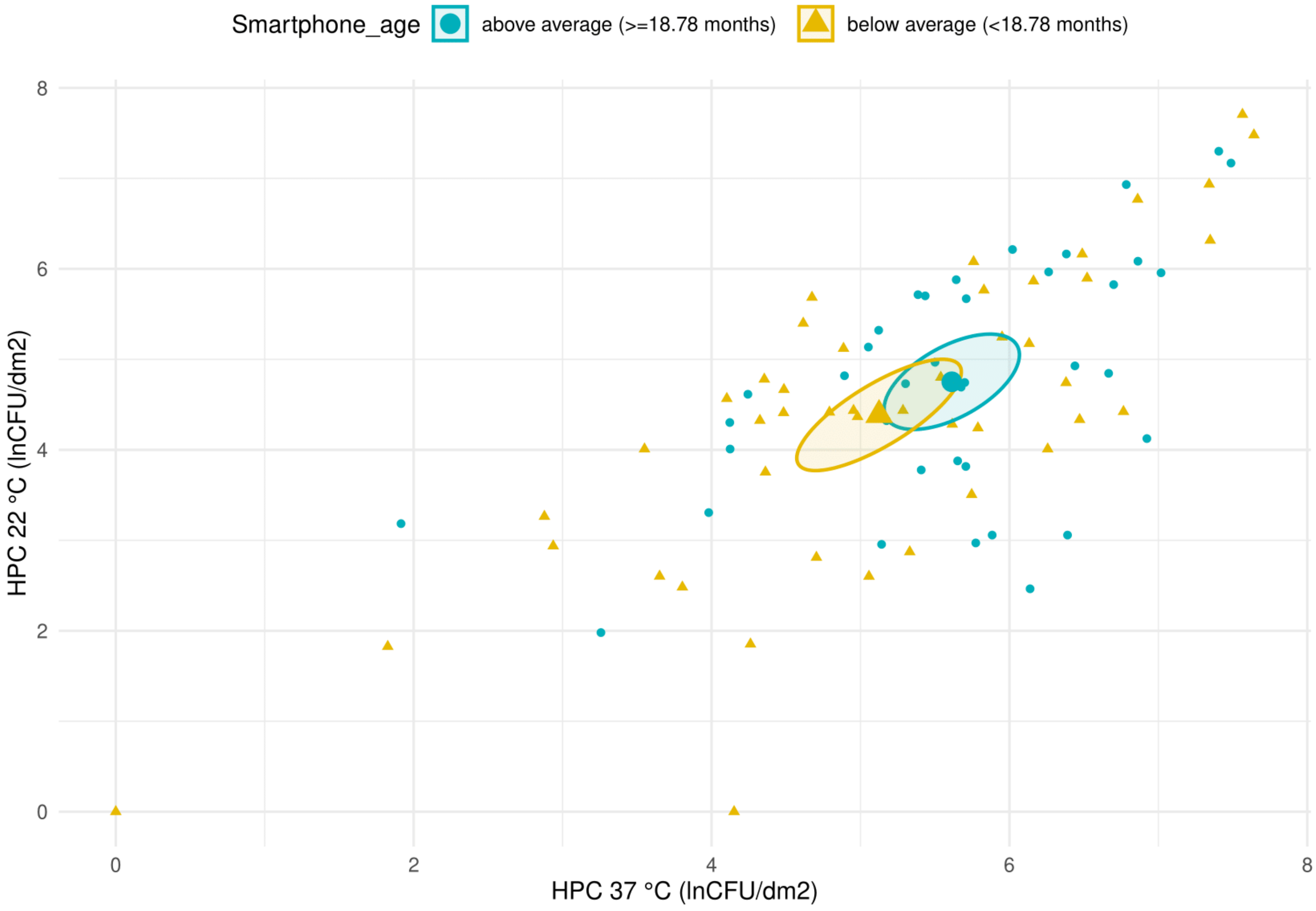

Figure S28: HPCs 37 °C and HPCs 22 °C based on Training frequency

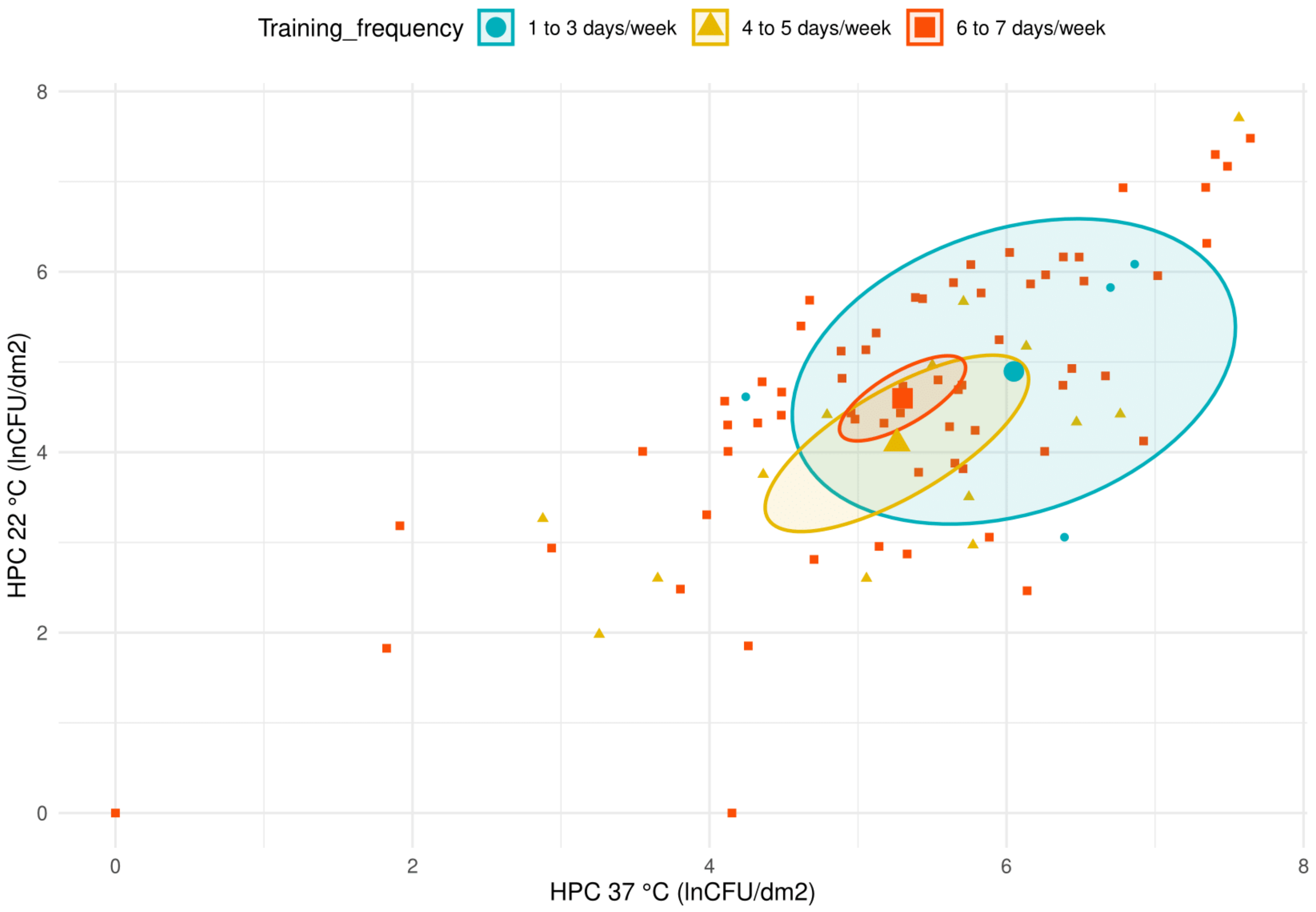

Figure S29: HPCs 37 °C and HPCs 22 °C based on Use with gloves

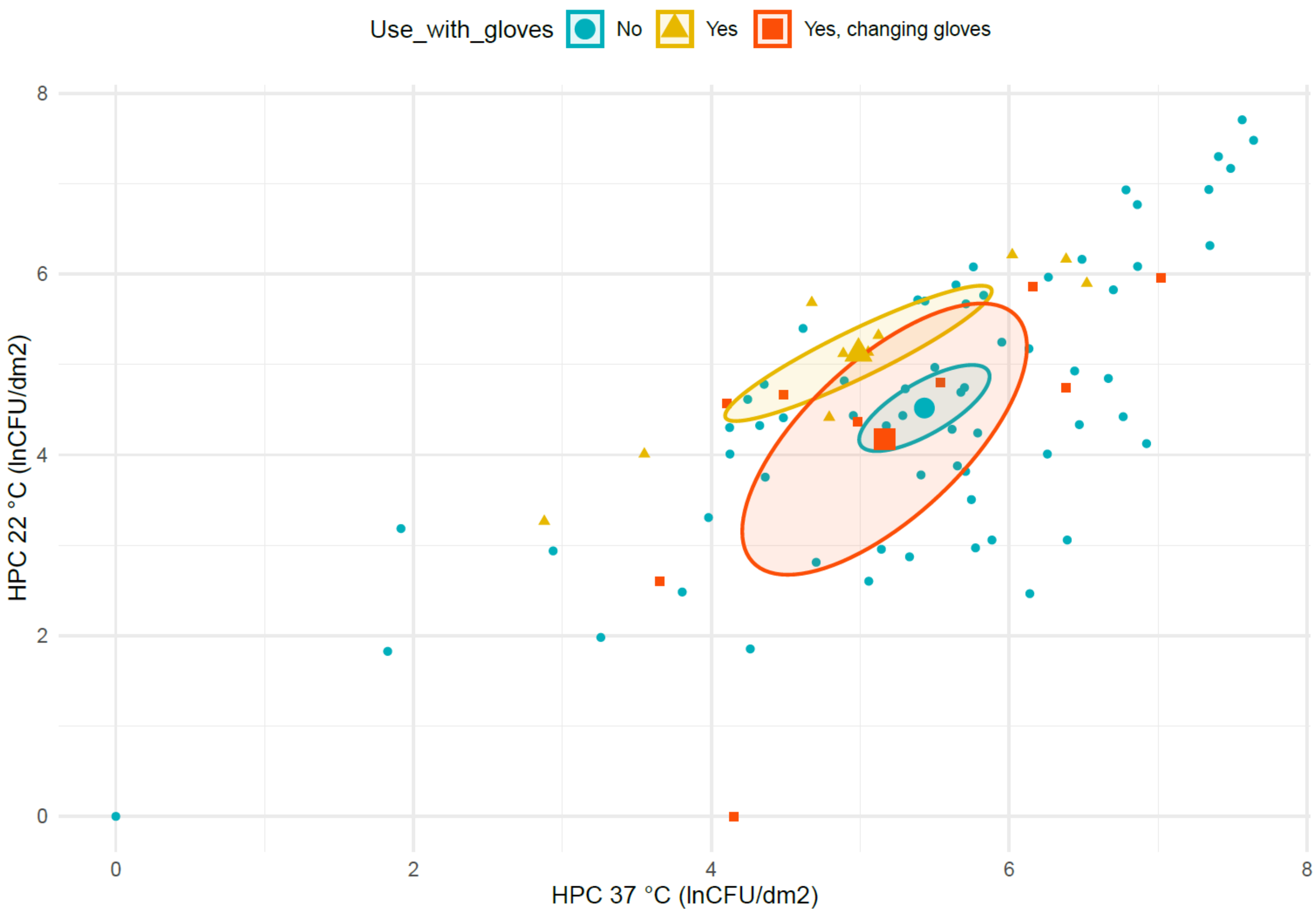

Figure S30: HPCs 37 °C and HPCs 22 °C based on Usual mean of transport

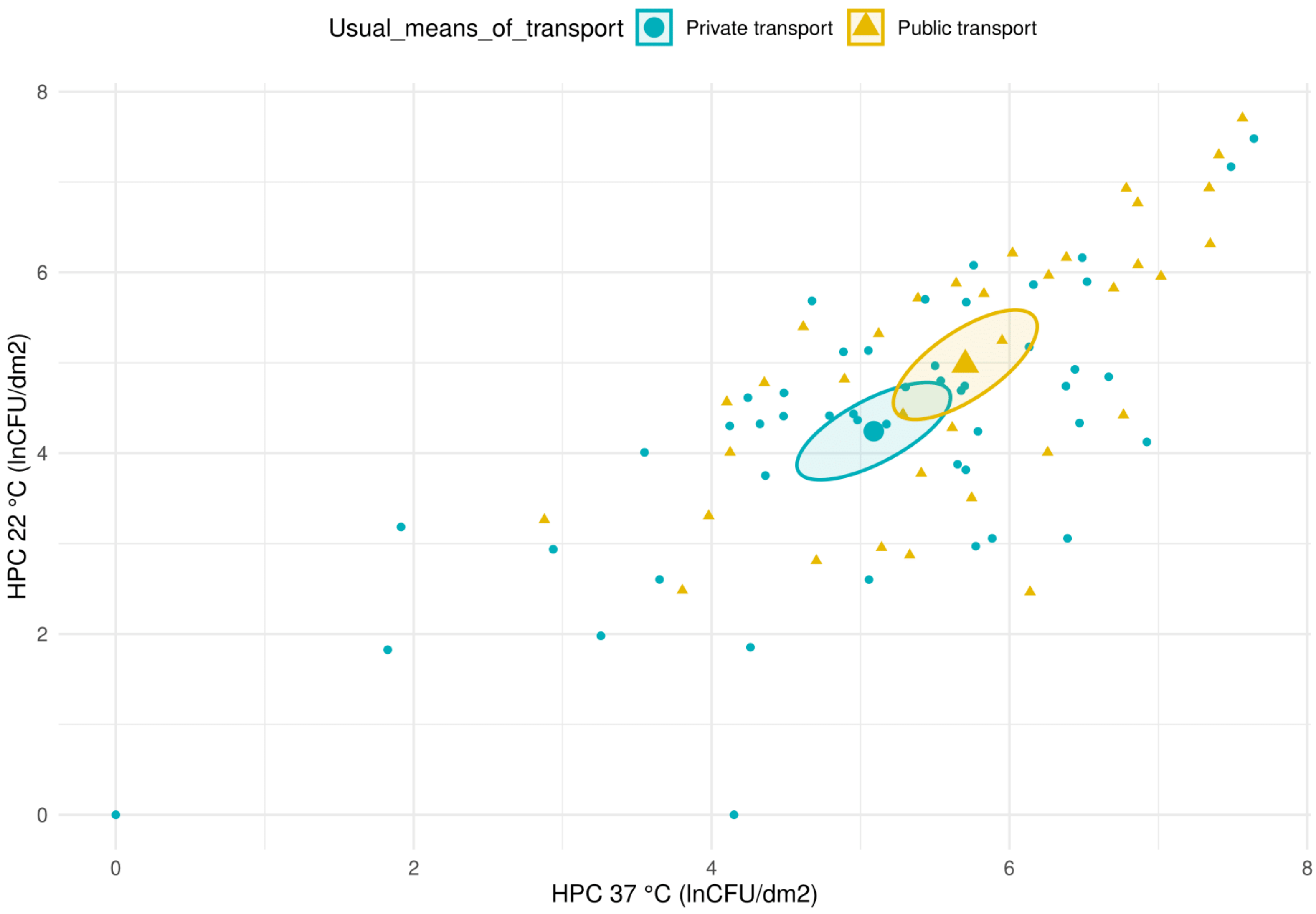

Scatter Plots comparing mean Staphylococci and Enterococci charges across the selected variables

Figure S31: mean Staphylococci and Enterococci charges based on Age

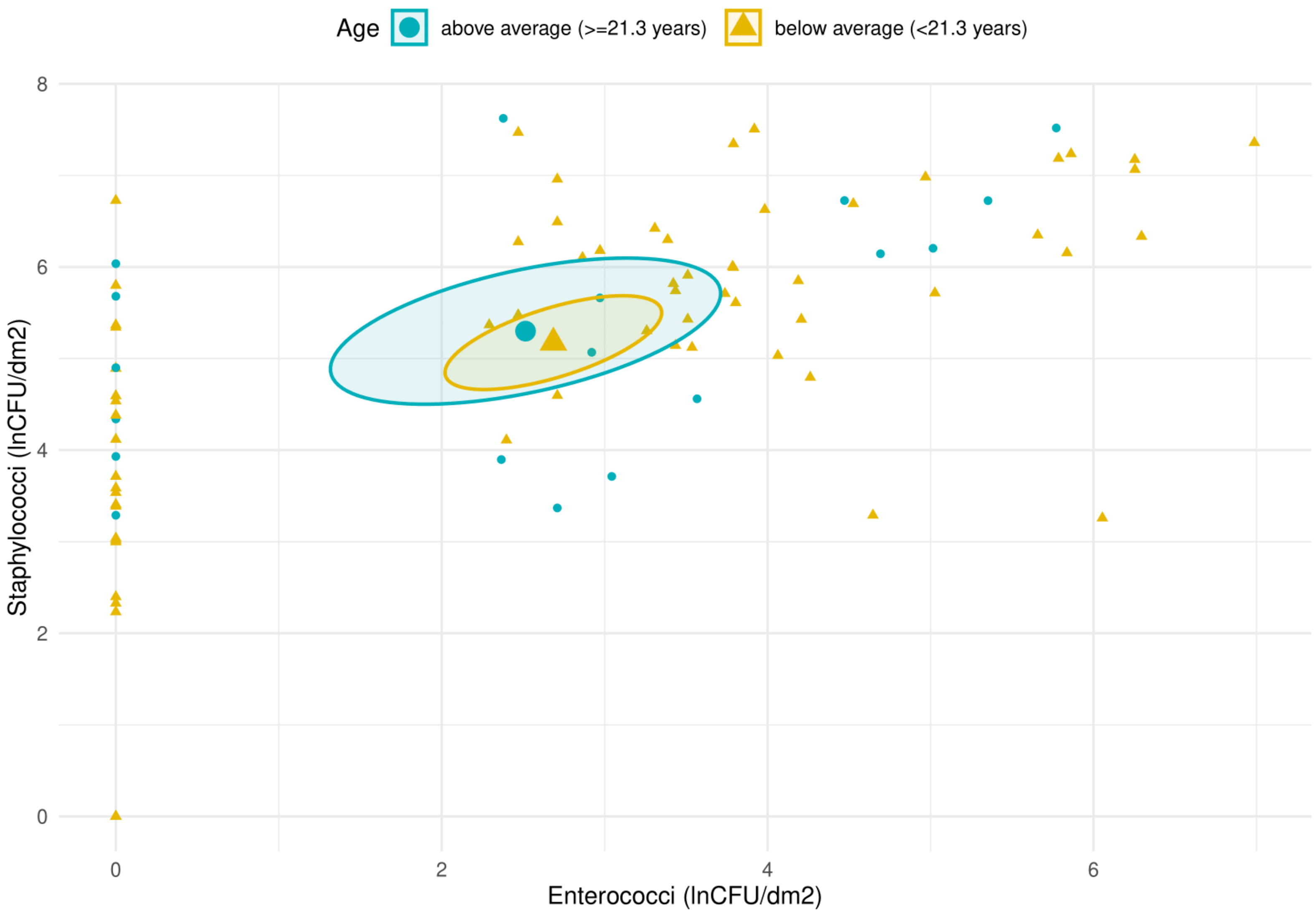

Figure S32: mean Staphylococci and Enterococci charges based on Cleaning frequency

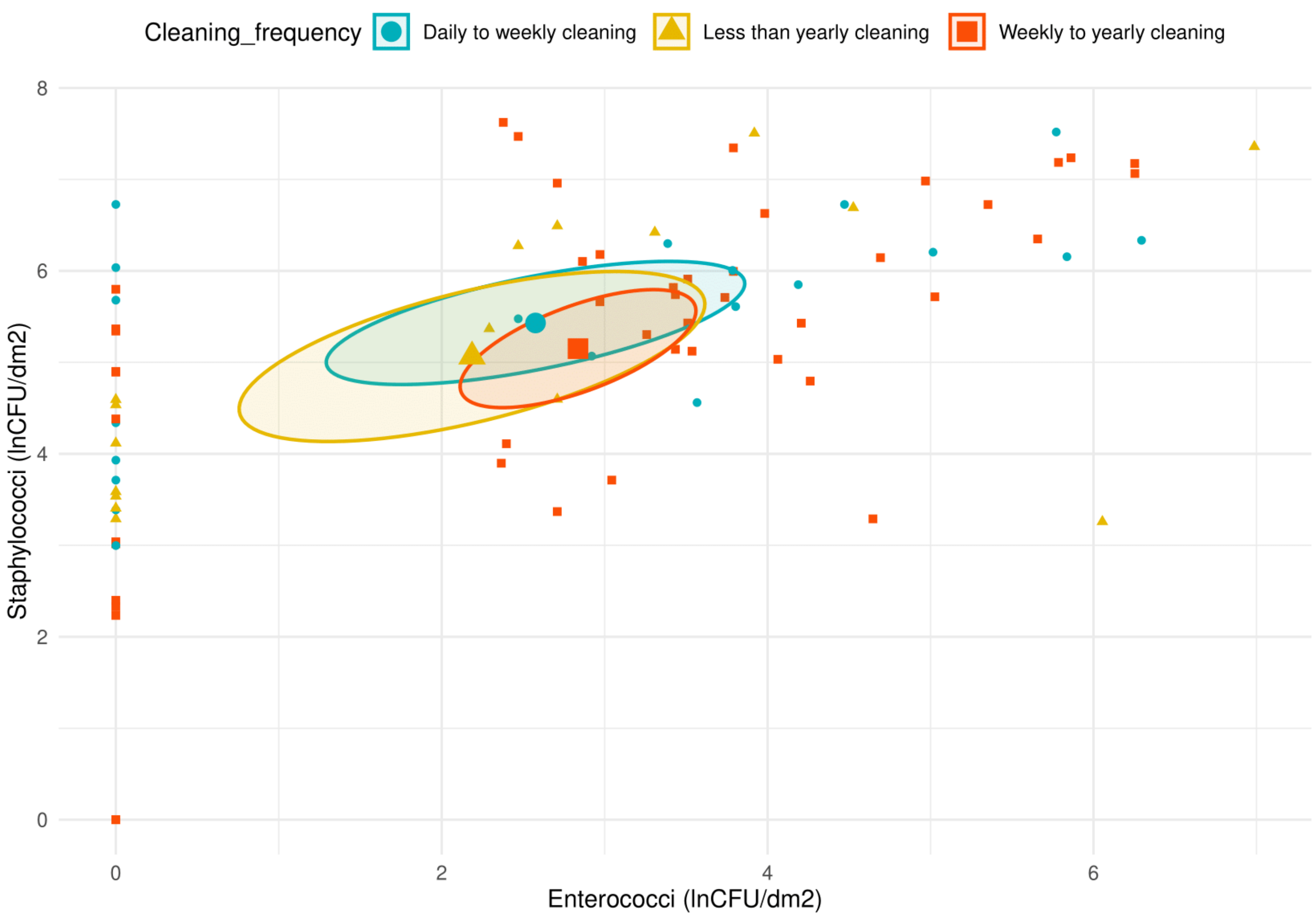

Figure S33: mean Staphylococci and Enterococci charges based on Cleaning method

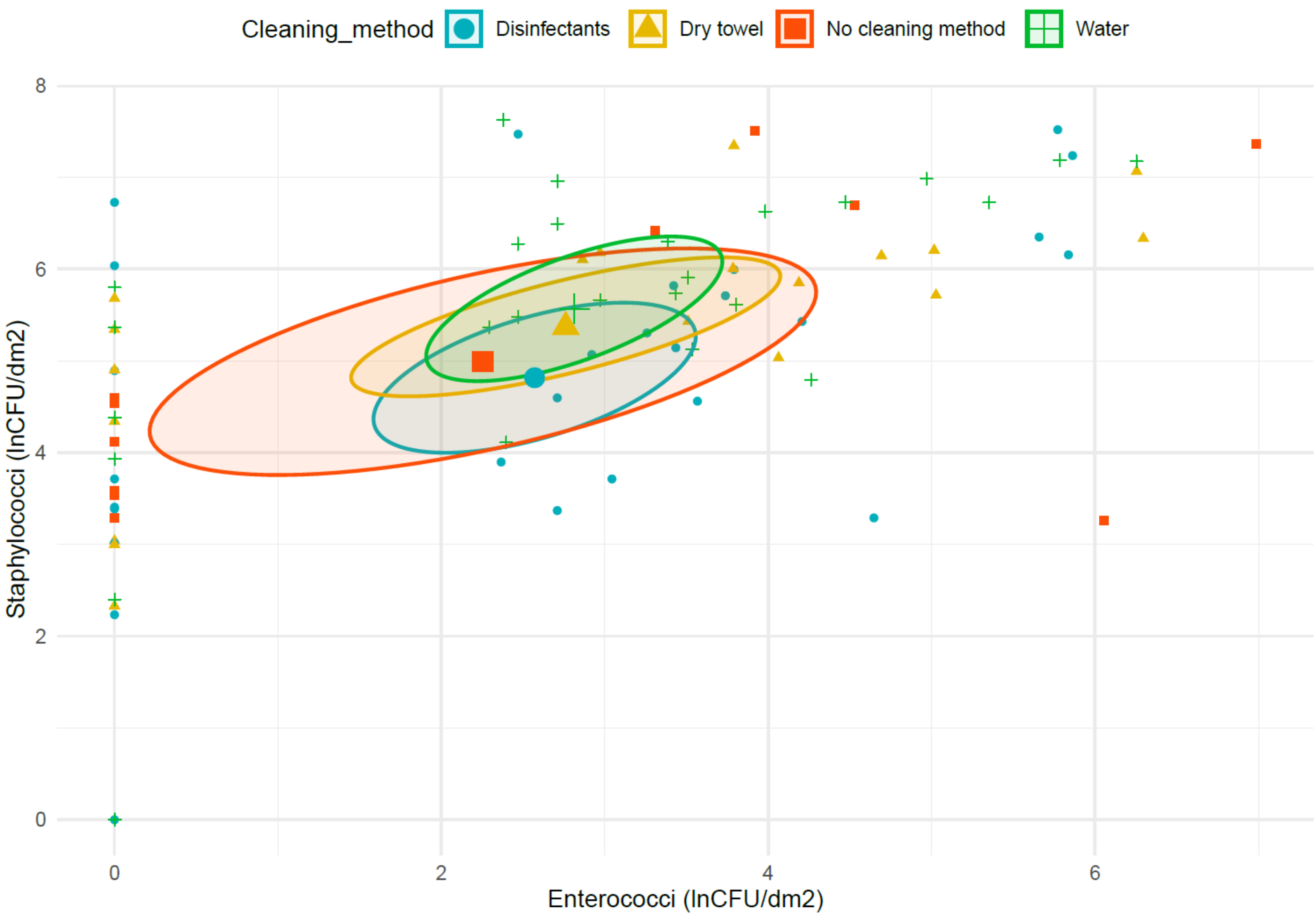

Figure S34: mean Staphylococci and Enterococci charges based on Cover type

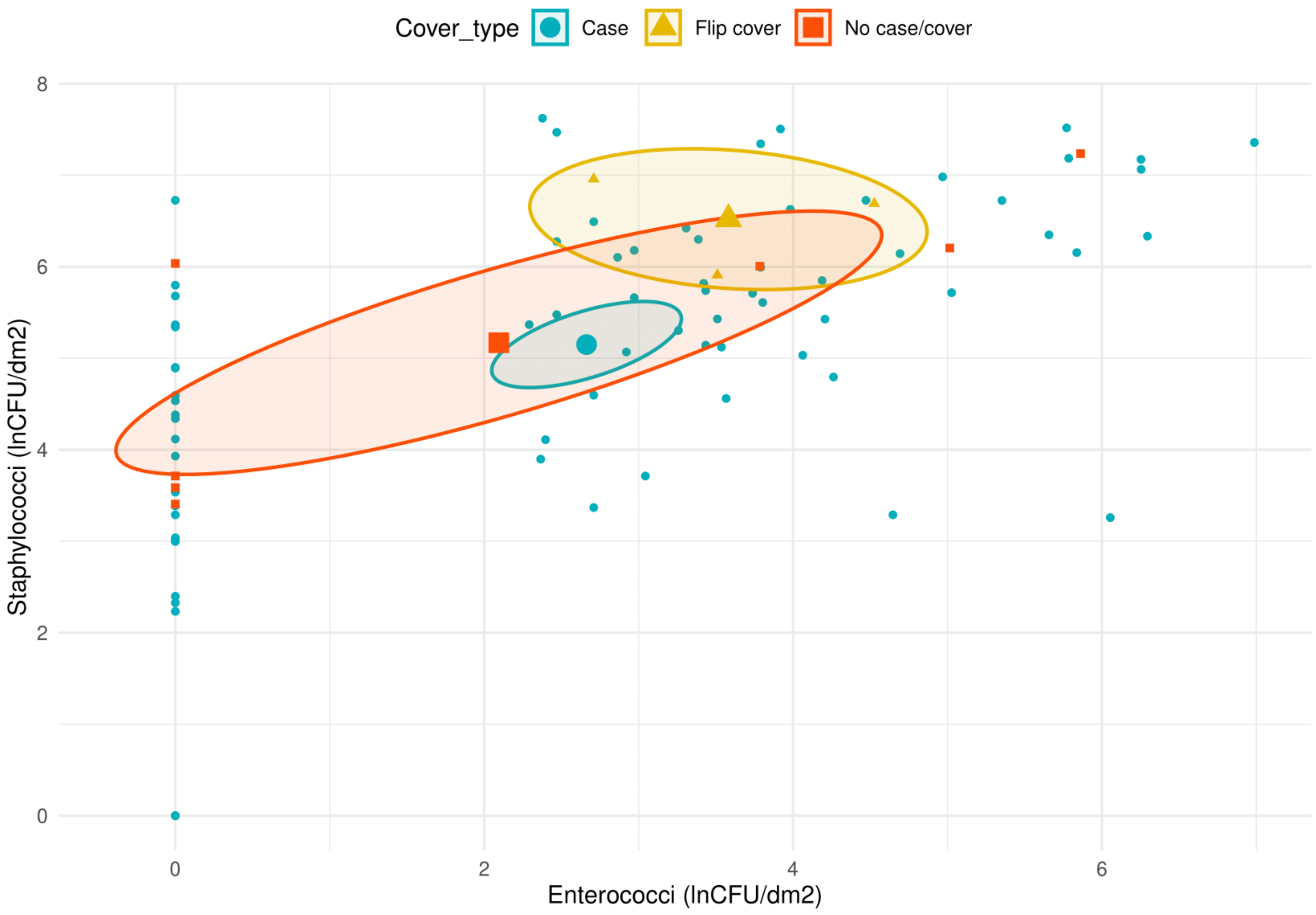

Figure S35: mean Staphylococci and Enterococci charges based on European Head SAR

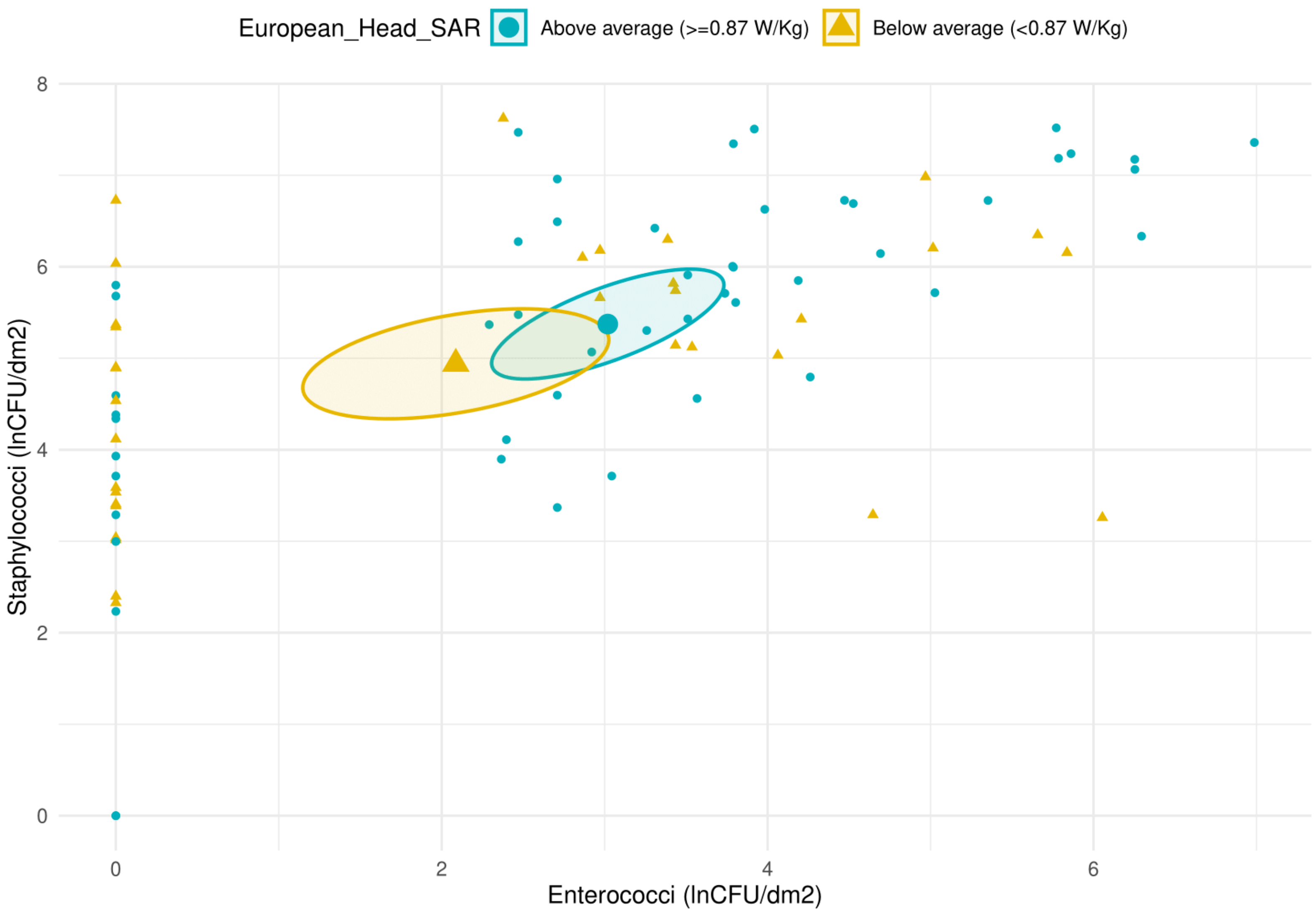

Figure S36: mean Staphylococci and Enterococci charges based on Gender

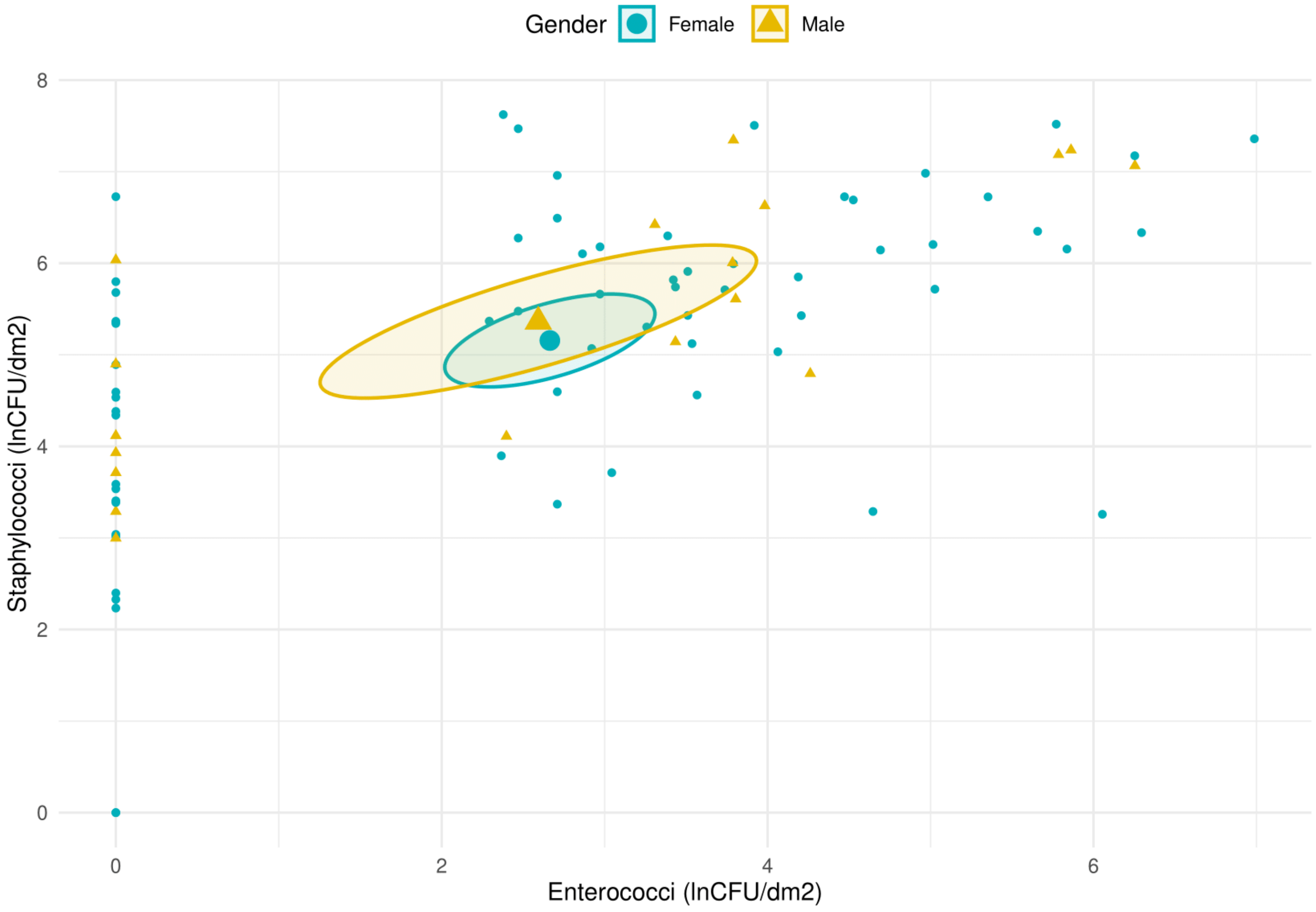

Figure S37: mean Staphylococci and Enterococci charges based on Health Status

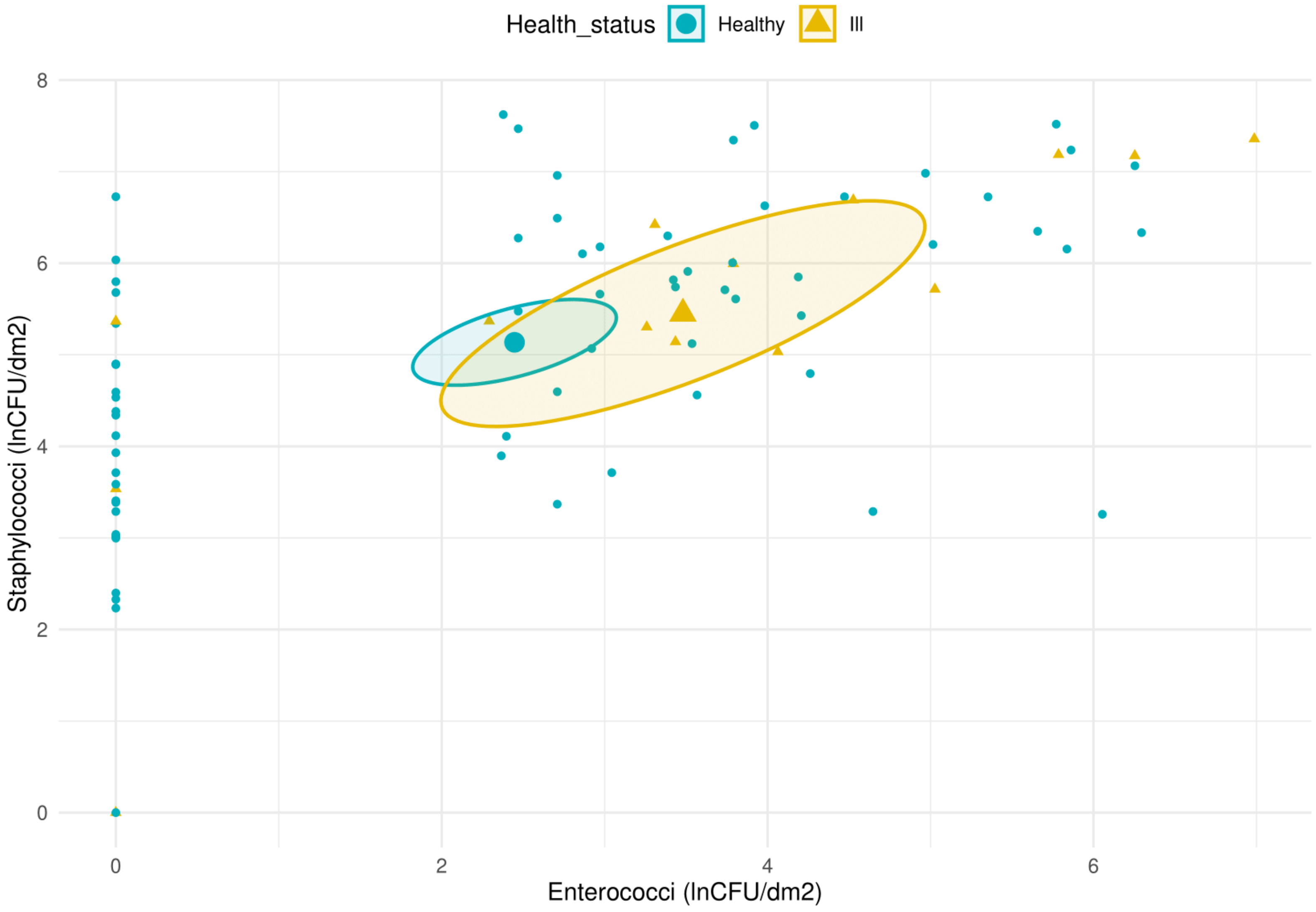

Figure S38: mean Staphylococci and Enterococci charges based on Last Cleaning

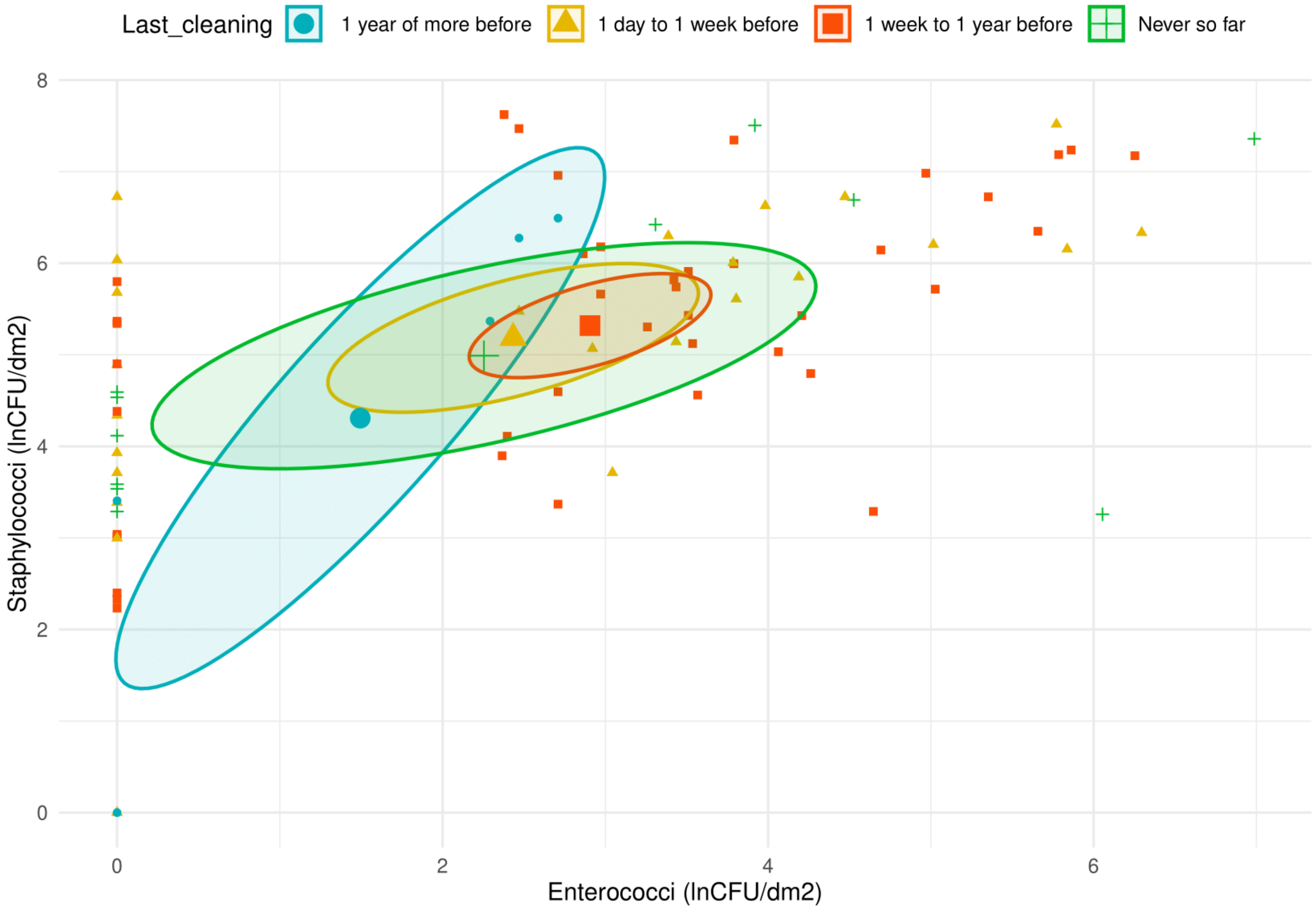

Figure S39: mean Staphylococci and Enterococci charges based on Means of transport in the day of sampling

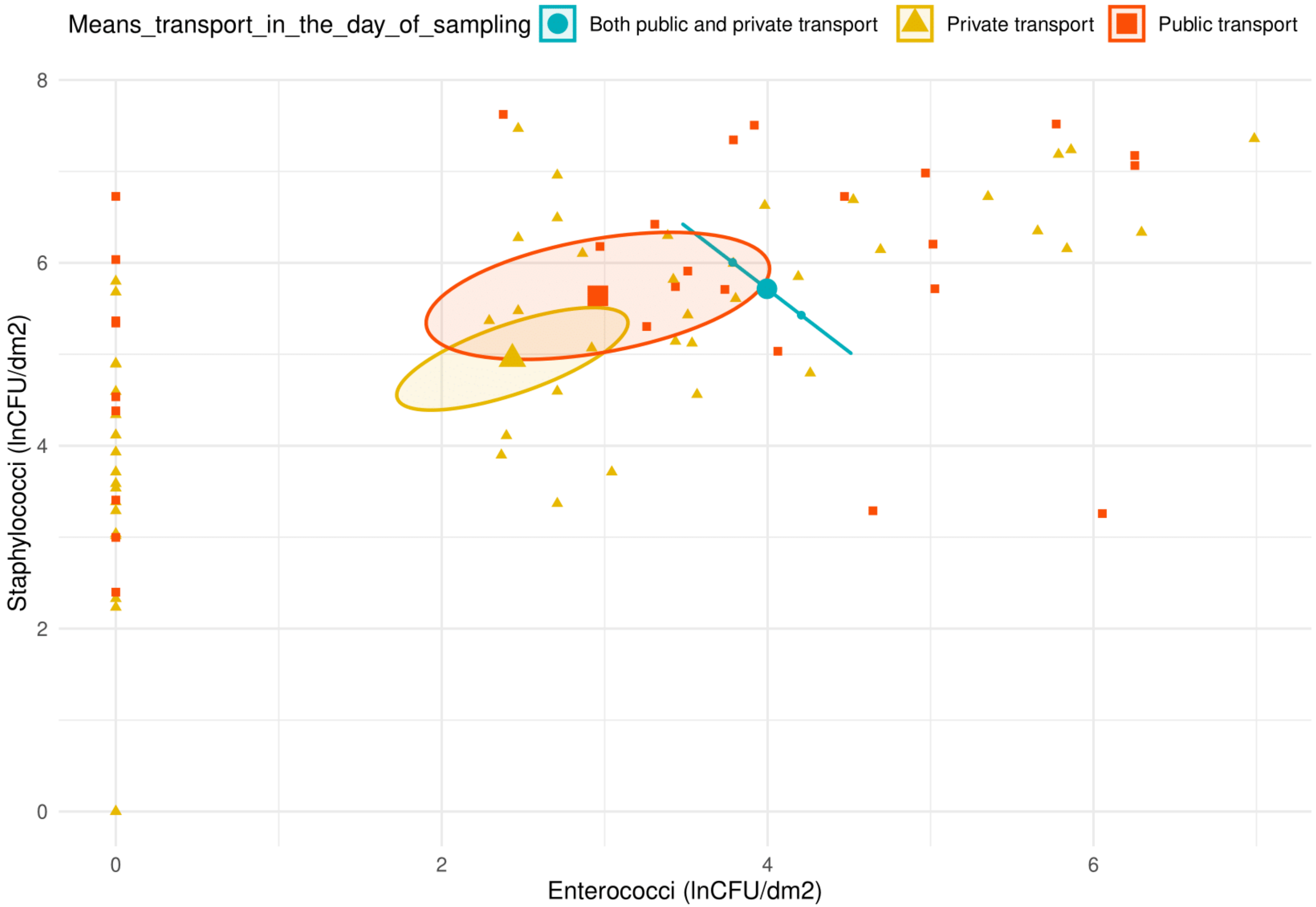

Figure S40: mean Staphylococci and Enterococci charges based on Place of training

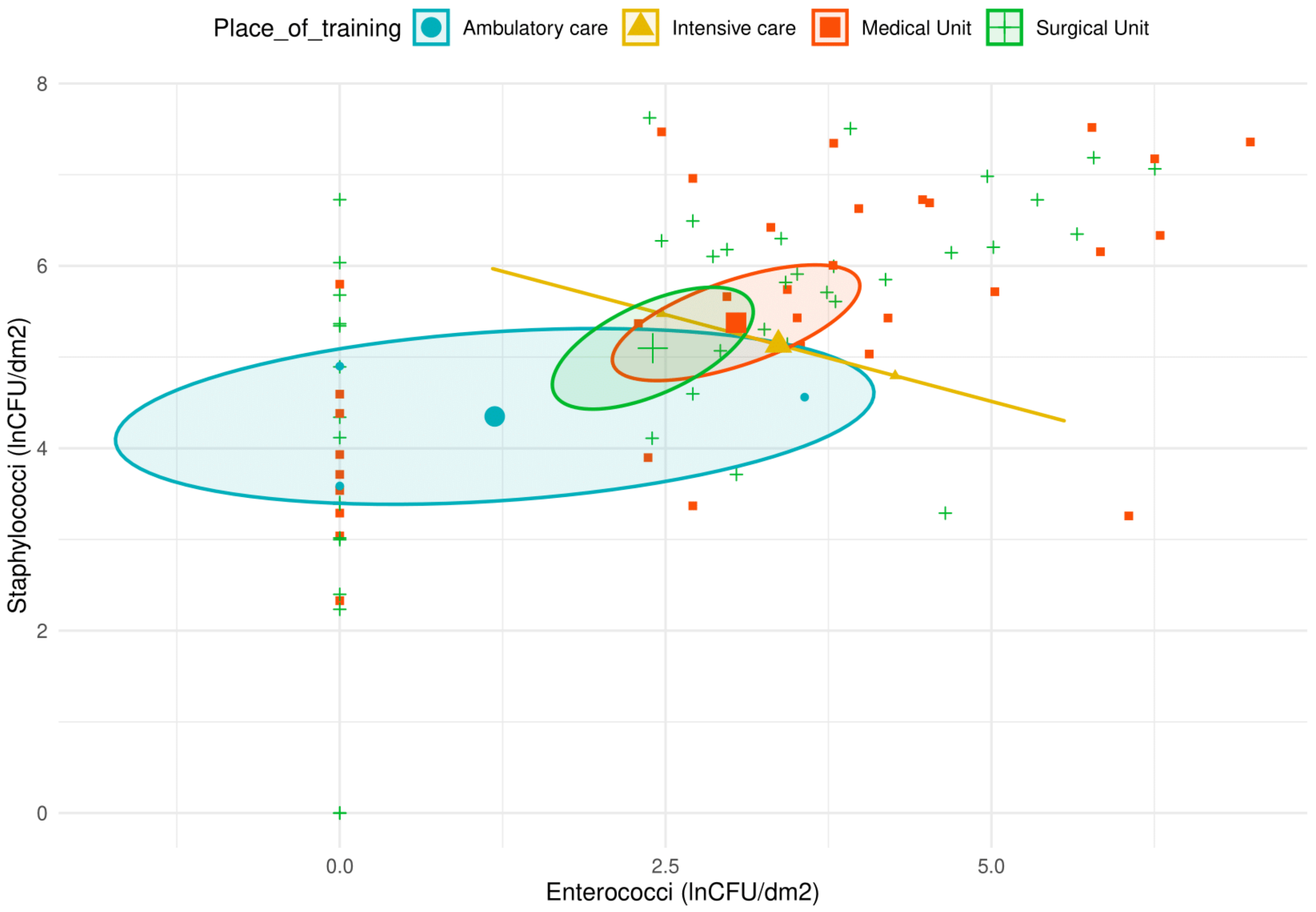

Figure S41: mean Staphylococci and Enterococci charges based on Screen protector

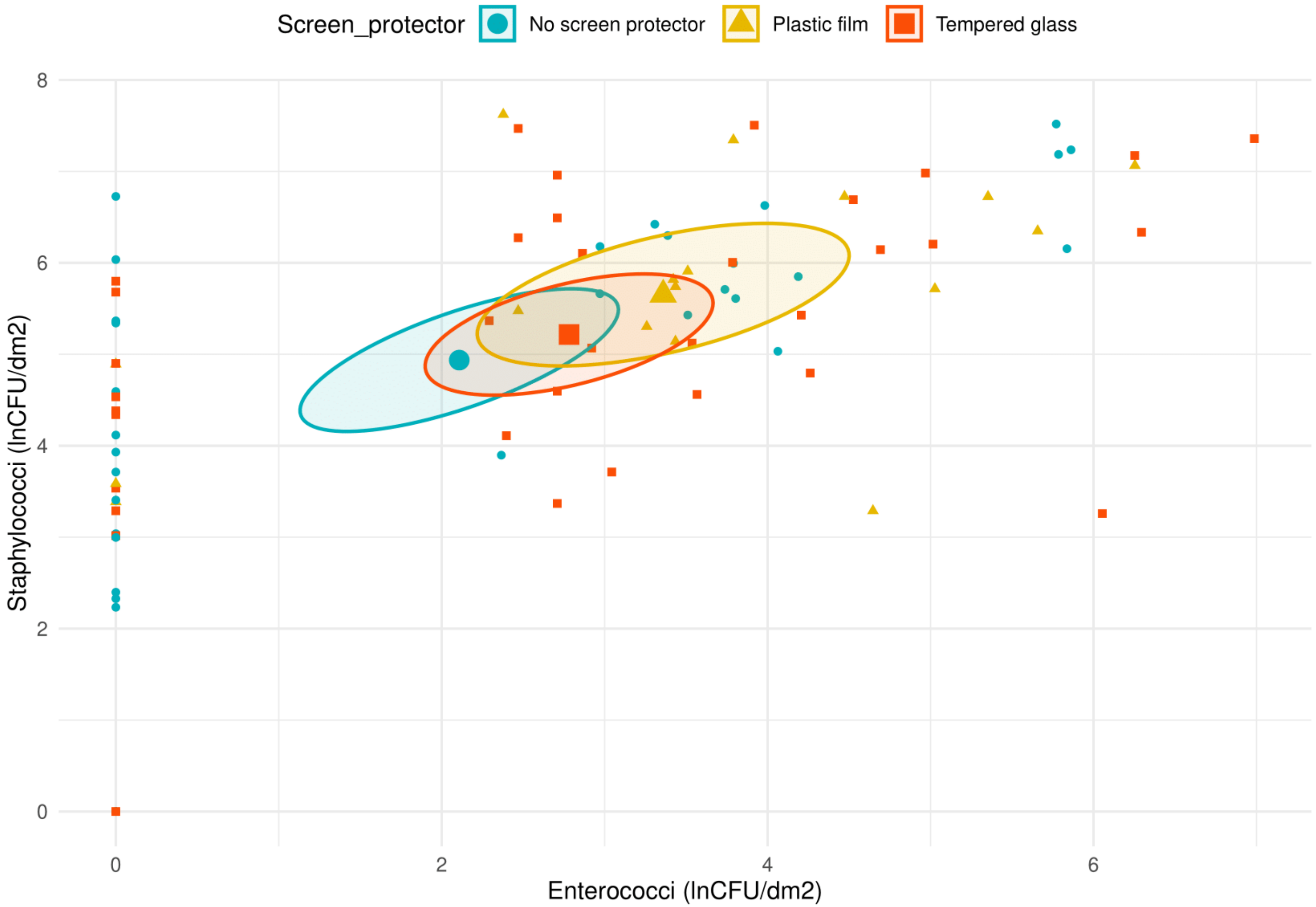

Figure S42: mean Staphylococci and Enterococci charges based on Smartphone age

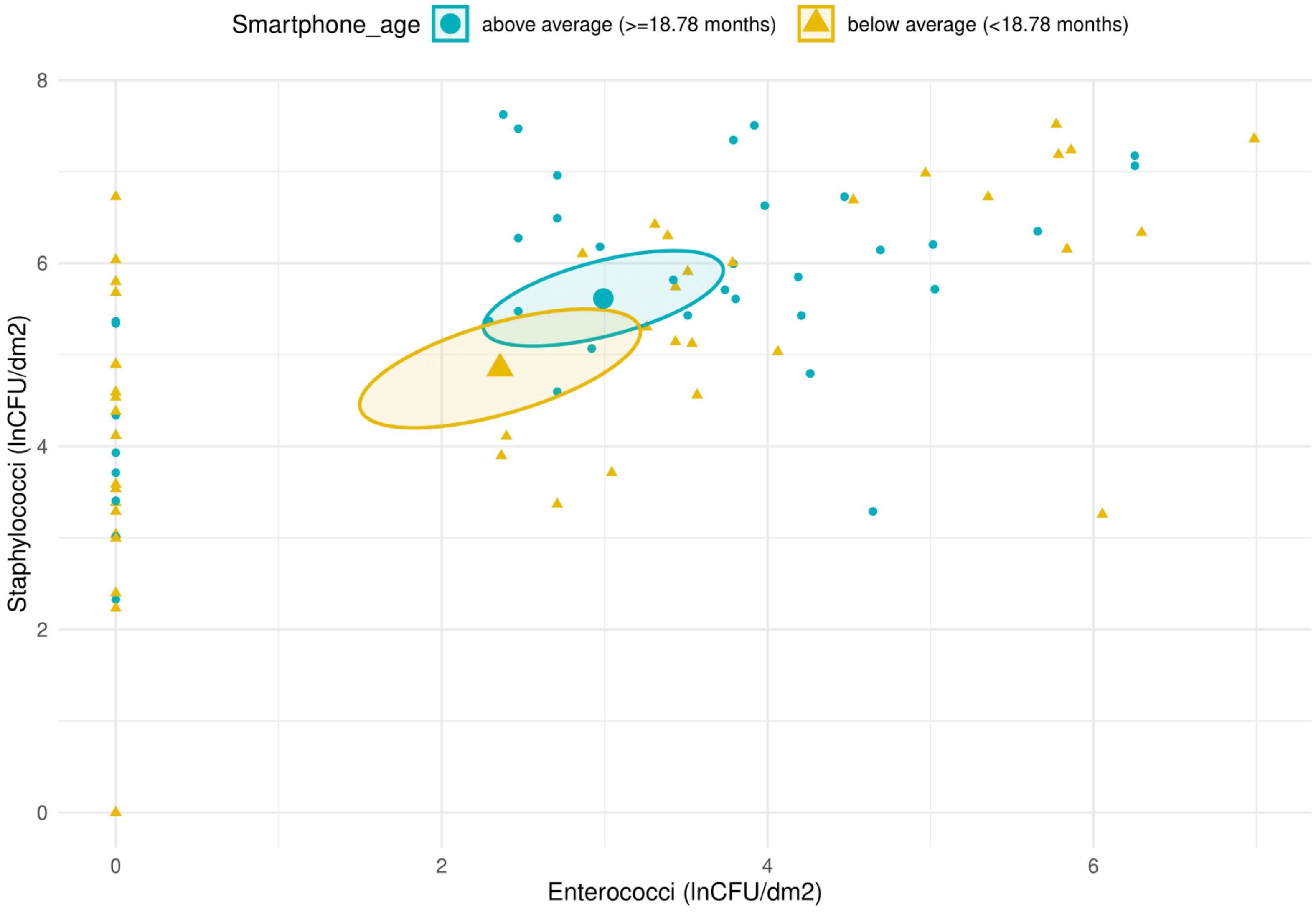

Figure S43: mean Staphylococci and Enterococci charges based on Training frequency

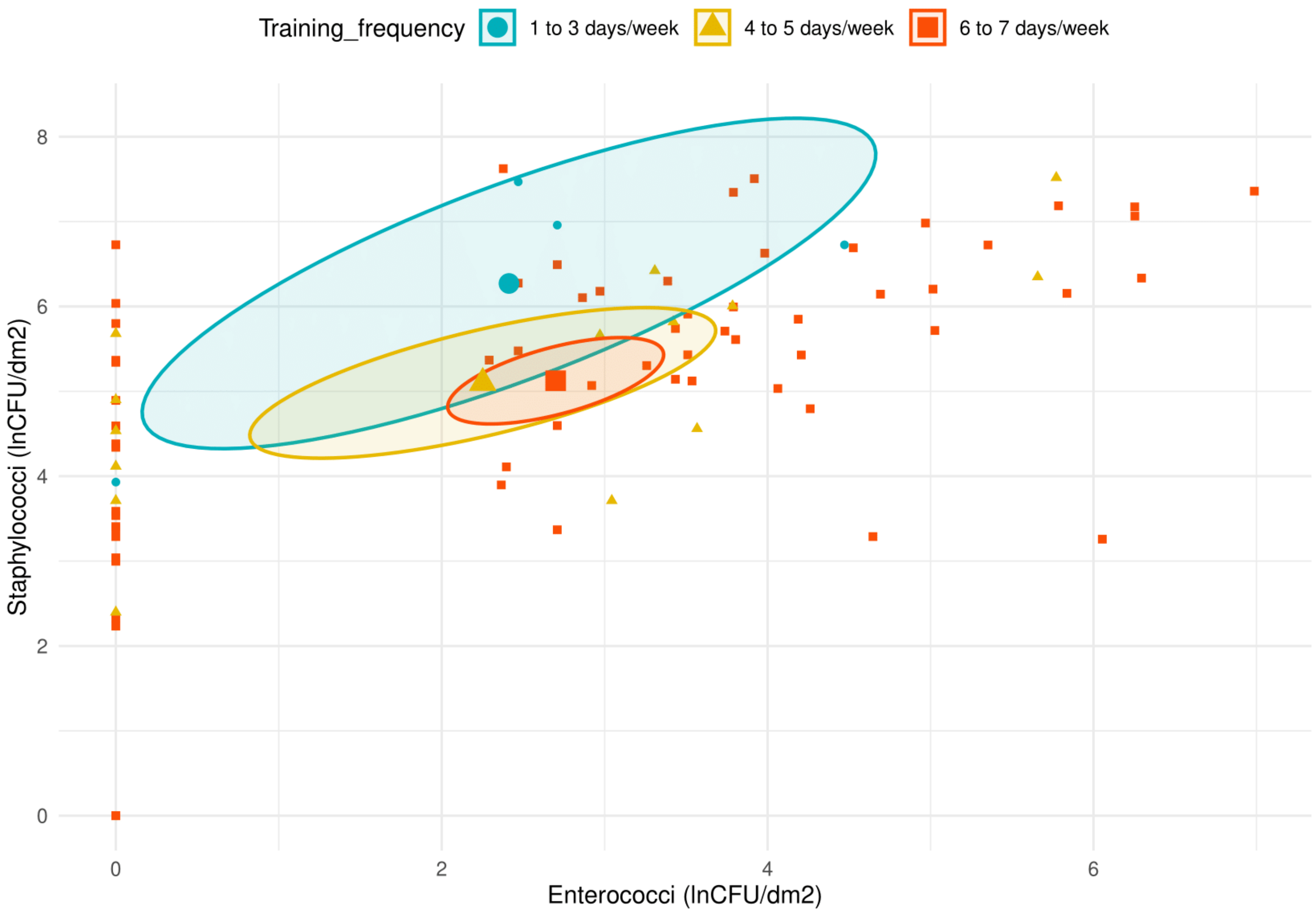

Figure S44: mean Staphylococci and Enterococci charges based on Use with gloves

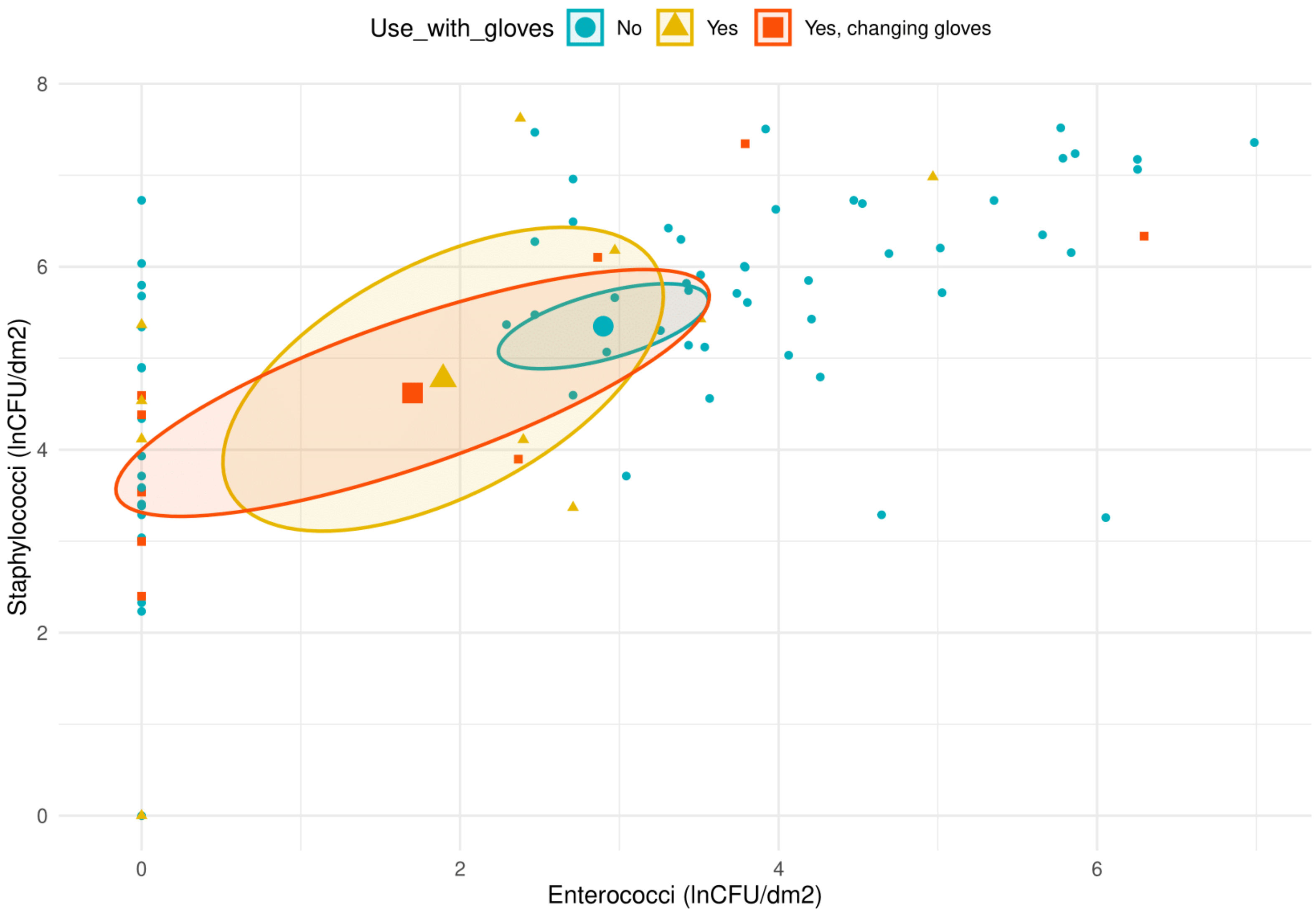

Figure S45: mean Staphylococci and Enterococci charges based on Usual mean of transport

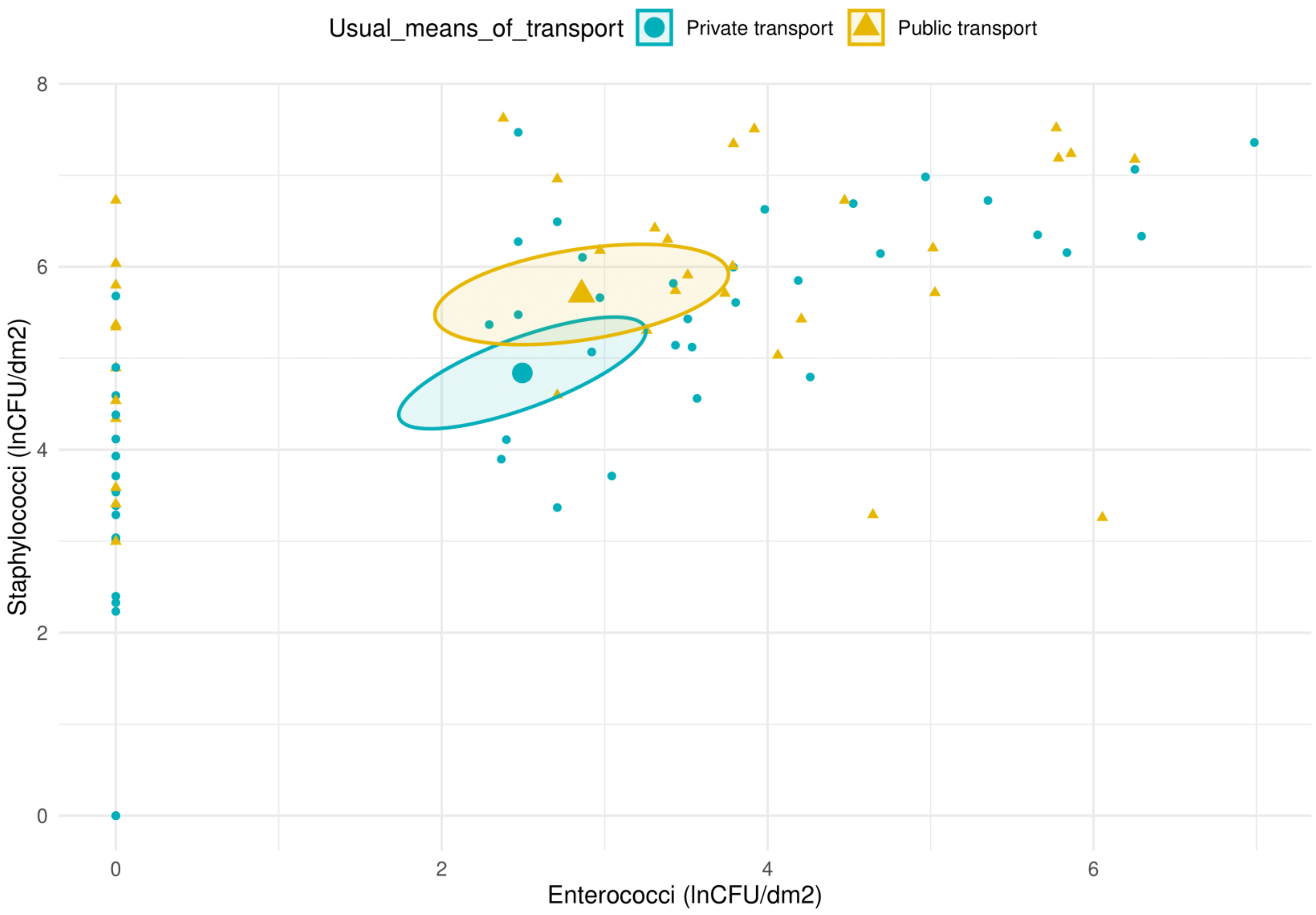

Supplement: Supplementary file 1 [file life-13-01349-s001.zip › life-2380619-supplementary.pdf]
